# Robustness and period sensitivity analysis of minimal models for biochemical oscillators

**DOI:** 10.1038/srep13161

**Published:** 2015-08-12

**Authors:** Angélica Caicedo-Casso, Hye-Won Kang, Sookkyung Lim, Christian I. Hong

**Affiliations:** 1 Department of Mathematical Sciences, University of Cincinnati, Cincinnati, OH 45221, USA; 2 Departamento de Matemáticas, Universidad del Valle, Cali, Valle, COL; 3 Department of Mathematics and Statistics, University of Maryland at Baltimore County, Baltimore, MD 21250, USA; 4 Department of Molecular and Cellular Physiology, University of Cincinnati, Cincinnati, OH 45267, USA

## Abstract

Biological systems exhibit numerous oscillatory behaviors from calcium oscillations to circadian rhythms that recur daily. These autonomous oscillators contain complex feedbacks with nonlinear dynamics that enable spontaneous oscillations. The detailed nonlinear dynamics of such systems remains largely unknown. In this paper, we investigate robustness and dynamical differences of five minimal systems that may underlie fundamental molecular processes in biological oscillatory systems. Bifurcation analyses of these five models demonstrate an increase of oscillatory domains with a positive feedback mechanism that incorporates a reversible reaction, and dramatic changes in dynamics with small modifications in the wiring. Furthermore, our parameter sensitivity analysis and stochastic simulations reveal different rankings of hierarchy of period robustness that are determined by the number of sensitive parameters or network topology. In addition, systems with autocatalytic positive feedback loop are shown to be more robust than those with positive feedback via inhibitory degradation regardless of noise type. We demonstrate that robustness has to be comprehensively assessed with both parameter sensitivity analysis and stochastic simulations.

Oscillatory systems are readily found in biology ranging from calcium oscillations (sec to min time scale) to circadian rhythms that recur daily (e.g. sleep/wake cycles). These enriched natural phenomena have been investigated mathematically, revealing theories behind these oscillators. Mathematical analyses indicate that a single time-delayed negative feedback loop or positive feedback mechanism is sufficient to create an autonomous oscillator^1^,^2^,^3^. Interestingly, molecular mechanisms of biological oscillators such as cell cycle and circadian rhythms contain both positive and negative feedback loops^4^,^5^. Recent efforts elucidate that a mechanism with both positive and negative feedback loops enhances chances for oscillations, and enables the system to vary the frequency without sacrificing the amplitude of oscillations^6^,^7^.

In this paper, we construct five simple models that generate autonomous oscillations and investigate their differences in dynamics and robustness in the context of period. These generic models are based on typical biochemical reactions such as transcription, translation, protein modification (e.g. phosphorylation), and degradation of molecular components along with regulatory processes for negative and positive feedback. These five models can be classified into two categories, two-variable and three-variable systems. Two-variable models include: (1) a reversible substrate-depletion oscillator, which is one of the most basic oscillatory mechanisms, (2) a negative and positive feedback loop via autocatalysis, and (3) a negative and positive feedback loop via inhibitory degradation. Three-variable models include: (4) a Goodwin oscillator that has a single negative feedback loop, and (5) a modified Goodwin model that incorporates an additional positive feedback loop. It is well studied that two-variable systems may have a stable steady state rather than sustained oscillations depending on the choice of parameter values^8^,^9^,^10^,^11^,^12^, while three- or more-variable systems can generate sustained oscillations more likely^8^,^10^,^12^,^13^,^14^,^15^. It is observed in many models that higher nonlinearity in kinetic equations can promote to generate sustained oscillations^10^,^14^,^15^,^16^,^17^. However, the high nonlinearity (or cooperativity) can be compensated by adding more variables. Kurosawa *et al.*^12^, for example, compared a three-variable Goodwin model with a four-variable Goodwin model and they showed that the increase in the number of variables, which may correspond to the additional protein modification, reduces substantially the degree of cooperativity.

Our bifurcation analyses of the five models show different period-determining parameters depending on the feedback mechanisms of the system, which create distinct dynamical behaviors and enlarged oscillatory regions with an additional positive feedback loop or a reversible reaction. In addition, we investigate the robustness of the period, which is critical in the case of circadian rhythms because the circadian clock needs to maintain its periodicity in the presence of various noises such as nutrient and/or temperature fluctuations^18^,^19^,^20^. There have been studies to investigate robustness and dynamical differences between different models in the context of period maintenance. For example, Wolf *et al.*^21^ investigated the period robustness of three different oscillatory systems (calcium oscillations, glycolytic oscillations, and circadian rhythms) with respect to 10% of parameter variations and showed that the system with negative feedback loop is more robust than the system with positive feedback loop in the context of period. Forger and Peskin^22^ considered a detailed model of mammalian circadian clock in the presence of molecular noise and investigated the dynamical differences in comparison to the corresponding deterministic model. Gonze *et al.*^23^,^24^,^25^ investigated robustness of a simple model against molecular noise and compared with its deterministic version of the model. Recently Gonze and Hafner^26^ showed that positive feedbacks enhance the robustness of the cell cycle with respect to molecular noise.

In this work, we apply two different types of noises, parameter variations and inherent stochasticity to the simple models above and identify the mechanisms that enhance the robustness of the system. We first use random perturbation of reaction rates of the deterministic differential equations, which corresponds to external perturbations such as temperature fluctuations. Then, we perform stochastic simulations to investigate period sensitivity in the presence of inherent noise based on the fact that each biochemical reaction is determined by molecular interactions, which are subject to intrinsic noise^27^,^28^. Both analyses reveal a group of robust models and less robust models, which will be discussed in Results and Discussion section. Some models are found to be fairly robust in both approaches. For example, a three variable model that incorporates both negative and autocatalytic positive feedback loops shows robust oscillations in both parameter sensitivity analysis and stochastic simulations. In contrast, some models demonstrate different robustness depending on the noise type and the strength of molecular noise. The substrate-depletion model with a single autocatalytic positive feedback loop shows robust oscillations in stochastic simulations, but not in parameter sensitivity analysis. There is also a model that is always sensitive to both random perturbation of parameter values and inherent noises. Furthermore, slight modifications of simple models are made to investigate how a small change in network topology affects the robustness of the system. Our simulation results suggest that two approaches, parameter random variations and stochastic simulations, are necessary to obtain comprehensive data for robustness and period sensitivity of the oscillatory system. In addition, the simulation results can be utilized to make informed decisions in implementing feedback loops to construct synthetic oscillators.

## Models and Methods

We consider five simple models that represent potential molecular mechanisms of biochemical oscillators and investigate the underlying features of the nonlinear dynamics exhibited by biologically relevant regulatory networks. Table 1 shows three two-variable systems and two three-variable systems. The table includes molecular wiring diagrams in the first column, corresponding systems of ordinary differential equations (ODEs) in the second column, and their numerical solutions in the last column. Molecular components, *M*, *P*, and *P*_*p*_ represent concentrations of mRNA, protein, and phosphorylated protein given in arbitrary units (a.u.), respectively. In wiring diagrams, solid lines represent biochemical reactions for production, degradation, or phosphorylation of molecules and dashed lines with arrow/blunt ends represent activation/inhibition regulatory processes. Here, *v* is the synthesis rate of mRNA given in arbitrary units per hour (a.u. per h), *η* and *k*_2_ are the synthesis rate of protein given in arbitrary units per hour and per hour (per h), respectively. All the other *k*_i_’s are the reaction rate constants whose units are per hour, *K*_*a*_ and *K*_*b*_ correspond to the thresholds of critical concentrations for inhibition and activation processes which are given in arbitrary units, and both *m* and *n* are Hill coefficients that represent the cooperativity of reaction kinetics. For each model, we perform extensive bifurcation analysis to find reasonable parameter spaces that will produce 22-hour oscillations. It is widely known that the dynamical behavior of oscillatory systems depends on wirings, choice of kinetic equations, and parameter spaces^12^,^29^,^30^,^31^. Therefore, comparing robustness of systems with different network topologies is a challenging task. In this report, we set our criteria to find a parameter set that can be varied at least by 40% for each model and performed our sensitivity analysis. In numerical solutions, curves in each model display the time evolution of each component of the model.

Wiring diagrams in Table 1 show a reversible substrate-depletion oscillator^3^ (Model 1); a negative-positive feedback loop via autocatalysis (Model 2); a negative-positive feedback loop with inhibitory degradation (Model 3); a Goodwin oscillator^1^ (Model 4); and a mixed model consisting of a Goodwin oscillator and Model 1 (Model 5). We carried out one- and two-parameter bifurcation analyses to investigate the dynamical change in the systems’ behavior^32^,^33^,^34^ (Supplementary Text S1). Numerical integration and bifurcation analysis of the deterministic models were performed via computer programs, XPP-AUTO and MATLAB. Stochastic simulations were performed on MATLAB.

## Results and Discussion

### Two-variable networks

The occurrence of biochemical oscillations requires two minimal conditions. The first condition is that the network must have at least two molecular components. In general, it is impossible to create oscillations with a single time-dependent component. The second condition for a two-variable system to undergo sustained oscillations is that the system must include an autocatalytic process in the molecular mechanisms. See Fall *et al.*^3^ for a mathematical analysis. It is important to note that the inclusion of a negative feedback loop in the system may lead to rhythmic behavior, but a two-variable system with negative feedback alone cannot generate sustained oscillations. An additional third component, an additional regulatory process such as an autocatalytic process, or a time delay is required in addition to a two-component negative feedback mechanism^3^,^8^. Based on these limitations, we investigate dynamical characteristics from three simple oscillatory mechanisms with two variables: a substrate-depletion oscillator with a reversible reaction and two negative feedback models with positive feedback either via autocatalysis or inhibitory degradation.

### Reversible substrate-depletion oscillator

Model 1, the first row of Table 1, displays a slight modification of a substrate-depletion oscillator which is one of the simple biochemical processes for sustained oscillations^3^. The molecular wiring, illustrated on the left panel, shows that protein *P* is produced at the constant rate of ***η*** and converted into phosphorylated protein *P*_*p*_ through an autocatalytic process. *P*_*p*_ is converted to *P* via a reversible reaction at the rate of *k*_6_. Both *P* and *P*_*p*_ degrade at the rates of *k*_3_ and *k*_5_, respectively. For exemplary phase analysis of Model 1, see Supplementary Text S2. This kind of autocatalytic process appears, for example, in glycolytic metabolic pathways^35^,^36^,^37^ and cell cycles^33^,^38^. As a specific example, a conversion of fructose-6-phosphate (F6P) into fructose-1,6-bisphosphate (FBP) is facilitated by an autocatalytic activation enzyme, phosphofructokinase, which results in glycolytic oscillations^3^.

**Figure 1:**
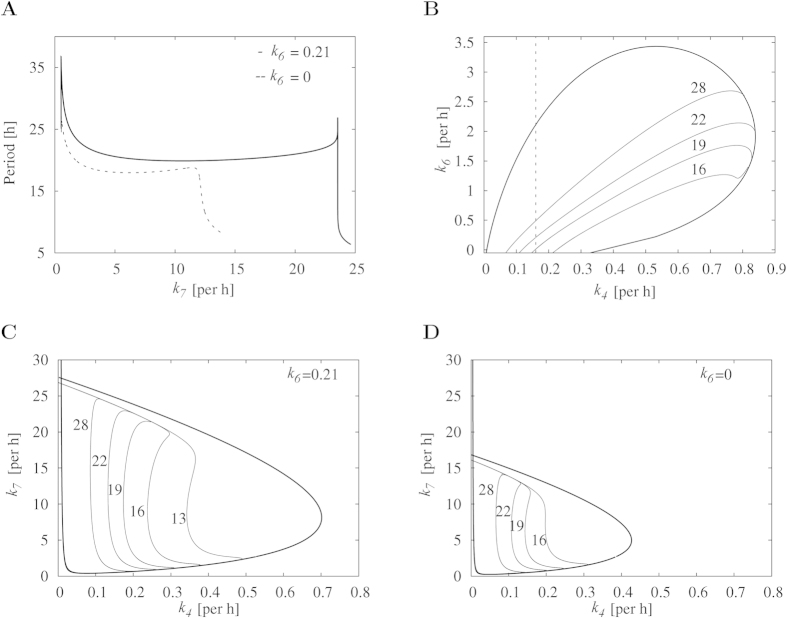
The effect of a reversible reaction on oscillatory behavior in Model 1. In (**A**), the period change is shown as a function of *k*_7_, the activation rate of autocatalysis, with or without a reversible reaction. The solid curve indicates the system with a reversible reaction when *k*_6_ = 0.21, and the dashed curve indicates the system without a reversible reaction when *k*_6_ = 0. The parameter value for *k*_4_ is drawn in dashed line in (**B**). In (**B**) a bifurcation diagram is shown for two parameters, *k*_4_ and *k*_6_. A region of oscillations is enclosed by the Hopf-bifurcation boundary. Each curve inside the boundary indicates a set of parameter values that produces a fixed period indicated on the curve. In the bottom panels, two bifurcation diagrams are displayed with a reversible reaction (**C**) and without a reversible reaction (**D**). The period of oscillations is given as a function of *k*_4_ and *k*_7_. The rest of the parameter values are taken from Table 1.

The key difference of Model 1 from a substrate-depletion oscillator is the inclusion of a reversible reaction from *P*_*p*_ into *P* (Table 1). To investigate the effect of this additional reaction on the system, we explore the behavior of the period of oscillations as each parameter varies. This reversible reaction in the system can be eliminated by simply setting *k*_6_ = 0. Figure 1 highlights the effect of a reversible reaction on the oscillatory behavior of the system by using three parameters, *k*_4_, *k*_6_, and *k*_7_. All the other parameter values are kept constant. Figure 1(A) shows the period change as a function of *k*_7_ with or without a reversible reaction, which demonstrates that the system with a reversible reaction almost doubles the region of oscillations. Figure 1(B) indicates the relationship between two parameters *k*_4_ and *k*_6_ in terms of the period, and shows that a larger region of oscillations can be achieved by choosing an appropriate value of *k*_6_. Each curve inside the Hopf-bifurcation boundary indicates a set of parameter values that produce periodic solutions with fixed period. Figure 1(C,D) compare two-parameter bifurcation diagrams in the presence (*k*_6_ = 0.21) or absence (*k*_6_ = 0) of a reversible reaction, which confirms that a region of oscillation can be enlarged when the reversible reaction comes into play in the dynamics. We wondered if we would continue to observe enlarged oscillatory domain with the reversible reaction using a different network topology. We extended Model 1 to incorporate a negative feedback loop (Model 1′) and demonstrate that the aforementioned reversible reaction enlarges oscillatory domain in most parameter space (Supplementary Text S3). Molecular mechanisms of cell division cycles involve an autocatalytic activation of the CycB/CDC2 complex, where CycB/CDC2 activates its own activator, CDC25. This activation of CycB/CDC2 is antagonized by WEE1 creating a meticulously controlled reversible activation and inactivation of the CycB/CDC2 complex^5^. Based on our results, we hypothesize that this reversible reaction not only controls the activation of CycB/CDC2, but it may also enlarge the region of oscillations.

### Negative-positive feedback loops

In this section, we investigate two minimal models consisting of both negative and positive feedback loops. These models, as illustrated in Table 1, have a negative feedback mechanism where the protein product inhibits its own transcription, and a positive feedback mechanism either via autocatalytic translation of the protein (Model 2) or via inhibition of protein degradation (Model 3). Model 2 is based on the results showing that FRQ protein promotes its own accumulation while forming a negative feedback loop inhibiting its own activator, WCC, in the Neurospora circadian clock^39^,^40^. Model 3 is based on the hypothesis that PER protein not only negatively inhibits its own activator, CLK/CYC, but it may promote its accumulation by inhibiting its own degradation in generating oscillations in the Drosophila circadian clock^41^. Models 2 and 3 have different network topologies, but both have regulatory mechanisms that allow nonlinear increase of protein abundance. In this regard, we consider that Models 2 and 3 have both negative and positive feedback loops except for the location of the positive feedback loop. Our bifurcation analyses of these two models demonstrate that their dynamical behaviors are substantially different.

**Figure 2:**
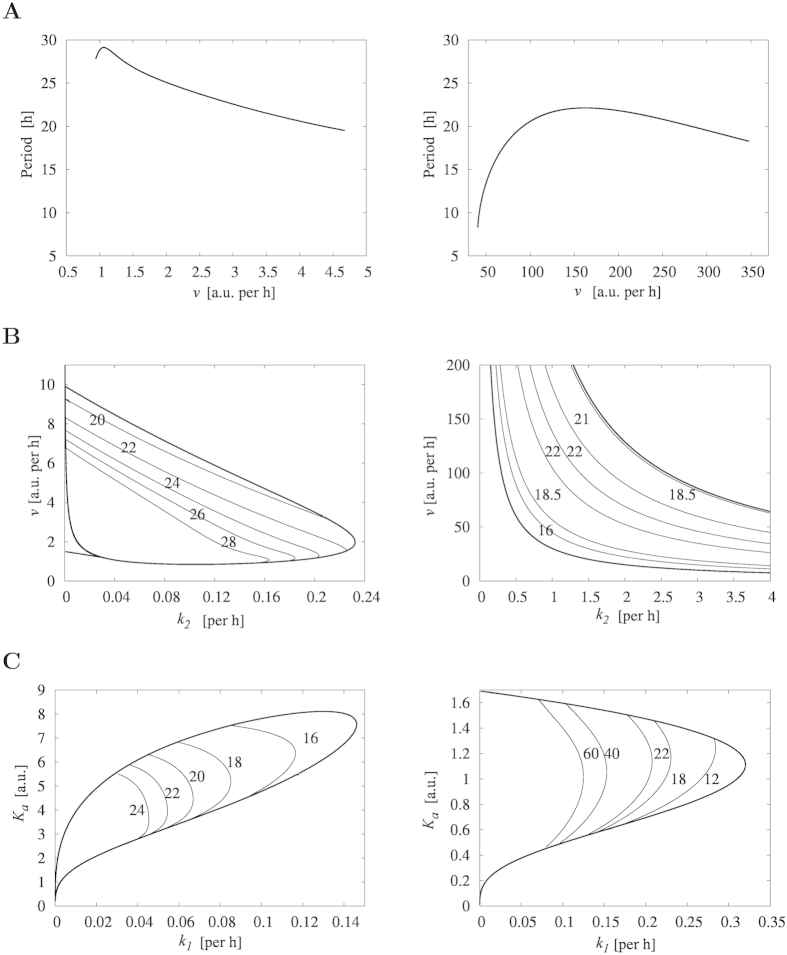
Bifurcation diagrams for Model 2 and Model 3. Left column displays bifurcation diagrams for Model 2. Right column displays bifurcation diagrams for Model 3. (**A**) Period as a function of the transcription rate of mRNA *v*. (**B**) Period as a function of *v* and *k*_2_, representing the transcription and translation rates, respectively. (**C**) Period as a function of *k*_1_ and *K*_*a*_, representing the degradation rate and the threshold of mRNA, respectively. All other parameter values are taken from Table 1. Numbers in (**B**) and (**C**) indicate the period of oscillations along each curve.

First, the patterns of periodic solutions are different between Models 2 and 3 (Table 1). Model 2 shows sinusoidal shapes of both *M* and *P*, and Model 3 shows distinct patterns of *M* and *P*, where *M* undergoes sharp rise followed by an exponential decrease due to the prolonged increase of *P* exerting negative feedback on the synthesis of *M*. Second, we observe drastically different behaviors of the systems with changes in parameter space. The top row of Fig. 2 displays bifurcation diagrams showing the period of oscillations as a function of *v*, which is the synthesis rate of mRNA in both models. As *v* increases, the period of Model 2 evolves with a small increase initially and then decreases monotonically (left panel in Fig. 2(A)), whereas the period of Model 3 increases at a relatively faster rate and then decreases at a slower rate (right panel in Fig. 2(A)). The behavior of Model 2 results from the autocatalytic effect on the protein. As *v* increases, increased mRNA results in reaching the threshold for autocatalysis faster, which leads to faster inhibition of mRNA synthesis resulting in decrease of period. In Model 3, the period initially increases as a function of *v*, because faster increase of mRNA leads to prolonged high level of protein due to its inhibitory role on its own degradation, which then leads to extended repression of mRNA synthesis, as indicated by the long exponential decay pattern of mRNA decrease. However, once it passes the peak period at *v* = 148, the period decreases with increasing *v*, because the trough of the protein level is above the threshold for the positive feedback to result in the observed sharp protein increase as shown in Table 1. Therefore, prolonged protein abundance actually decreases when *v* > 148, resulting in a shorter duration of negative feedback. See Supplementary Information for numerical solutions at different values of *v* (Supplementary Fig. S1).

Lastly, we observe both similarities and differences in two-parameter bifurcation diagrams between Models 2 and 3 (Fig. 2(B,C)). Regions of oscillation are bounded by the outer loop of Hopf bifurcations, and each curve inside the oscillatory regions shows a collection of two parameter values that produce limit cycles with a fixed period. The period undergoes mostly monotonic decreases as a function of both *v* and *k*_2_ in Model 2 (left panel in Fig. 2(B)), but non-monotonic changes of period are observed in Model 3 (right panel in Fig. 2(B)). On the other hand, we observe some similarities as a function of *k*_1_ and *K*_*a*_, where *k*_1_ represents the degradation rate of mRNA and *K*_*a*_ represents the critical concentration for inhibition of mRNA transcription, respectively. For example, the period decreases with an increasing *k*_1_ in both models, while *K*_*a*_ is held fixed (Fig. 2(C)). However, we also observe some differences such as the boundary of oscillations. The above simulations demonstrate that two different types of positive feedback mechanism result in different phenotypes in the nonlinear dynamics. In addition, Model 2 is dramatically more robust than Model 3, indicating that positive regulation via autocatalysis may play a key role in the dynamics to maintain robustness of the system. This will be discussed in Robustness and period sensitivity analysis section below.

### Three-variable networks

In this section, we investigate two molecular wiring diagrams described by three molecular components: a Goodwin oscillator, which has a single negative feedback loop, and a model that incorporates autocatalysis with a reversible reaction in addition to the Goodwin oscillator as described in Table 1. The negative feedback regulation is essential in generating autonomous oscillations in circadian rhythms^42^,^43^,^44^. However, molecular mechanisms of circadian rhythms consist of multiple feedback loops including both positive and negative feedbacks^45^. We explore dynamical differences between the Goodwin oscillator and a model that incorporates both negative and positive feedbacks.

In the 1960’s, Goodwin^1^,^46^ proposed the first mathematical model of a biochemical oscillator based on negative feedback alone. Since then, many scientists have revisited and studied Goodwin’s oscillator in many different contexts such as circadian clocks^47^,^48^, HES1 oscillator in the embryo^49^ and other theoretical approaches^8^,^50^,^51^,^52^,^53^,^54^. For example, a Goodwin oscillator was recently used to investigate Hill coefficient of HES1 oscillator that plays a critical role during somitogenesis^49^. HES1 forms a negative feedback loop, and Zeiser *et al.*^49^ assumed intermediate components of HES1 (e.g. pre-mRNA, mRNA, etc.) in order to explore modifications of the Goodwin oscillator. The molecular wiring of a Goodwin oscillator is presented in Table 1. Here, mRNA (*M*) is translated into protein (*P*), and this protein is transformed into the end product (*P*_*p*_), which inhibits the transcription of mRNA. Note that *P*_*p*_ is not reversible in this model. Parameters *v*, *k*_2_ and *k*_4_ determine the rates of transcription, translation, and protein modification, respectively. Parameters *k*_1_, *k*_3_, and *k*_5_ are degradation rates for each component. As previously shown, the period of oscillations is sensitive to the rate of degradation of each component, but not to the rate of synthesis^47^.

We then extend the Model 4 to incorporate an autocatalytic process with a reversible reaction, which is a combination of a Goodwin oscillator and Model 1 (Model 5, Table 1), and investigate dynamical differences between the Goodwin oscillator and Model 5. Model 5 can also be considered as an extension of Model 2, which describes the activator-inhibitor oscillator. Our simulation results show that all parameters except one maintain the identical features of the Goodwin oscillator in the context of period variations, which are: (1) the period decreases as a function of degradation rates of all three components, and (2) the period is insensitive to the change of synthesis rates of mRNA and protein. In contrast, the rate of protein modification (e.g. phosphorylation), *k*_4_, becomes a sensitive parameter in Model 5 (Supplementary Fig. S2). Our simulations also demonstrate that the region of oscillatory domain is enlarged in the presence of a reversible reaction similar to the results from Model 1 (Data not shown).

### Robustness and period sensitivity analysis

Circadian rhythms are known to exhibit robust periods in response to external and internal variations, including temperature fluctuations and molecular noise, respectively. Along with the phase of the rhythm, the period is a key characteristic of biological oscillators. The robustness of the circadian period has been extensively investigated. For example, temperature and nutrient compensation (i.e., maintenance of the circadian period in different temperature or nutrient conditions) is widely observed in various organisms from lower eukaryotes to mammalian systems^18^,^19^,^20^.

In this section, we investigate the robustness of the period from the five models described in the previous sections. The period sensitivity analysis is carried out using two different approaches. The first approach is to use random perturbations of parameter values in the deterministic differential equations, which corresponds to external noise. The other approach is to use stochastic simulations of the models in which inherent stochastic noise is taken into account. The investigation of period robustness may identify which molecular mechanisms help to maintain the period of oscillatory systems in the presence of internal or external perturbations. Furthermore, minimal networks that are relatively insensitive to noises may become building blocks of more complicated oscillatory systems.

#### Parameter random perturbations

External fluctuations such as temperature alter the chemical reaction rates in biological systems. However, systems maintain their robustness within a certain range of perturbations. To test the robustness of period in the models given above, we consider random changes in parameter values of each model.

Here we assume that all parameters corresponding to reaction rate constants follow normal distributions with mean, *μ*, defined as the default value of each parameter given in Table 1, and standard deviation, *σ*, defined as 0.06 *μ*. This implies that random perturbations of each parameter lying within *μ* ± 0.18 *μ* are included in approximately 99.7% of the normal distribution. Note that all Hill coefficients and critical thresholds are fixed throughout simulations in this section. A total of 4000 random perturbations for each model are made to verify the sensitivity of period to parameter perturbations. It is worth mentioning that as σ decreases, the pattern of the distribution of histograms becomes more concentrated around the mean value, which leads to similar and narrow histograms among models. Therefore, the comparison of histograms with smaller σ does not provide the much distinction among models. In order to justify the choice of the sample size of 4000, we have measured mean and standard deviation of period distribution in each model as a function of sample size (Supplementary Fig. S3). It is observed that the mean and standard deviation of periods within each model remain almost the same as the sample size varies.

**Figure 3:**
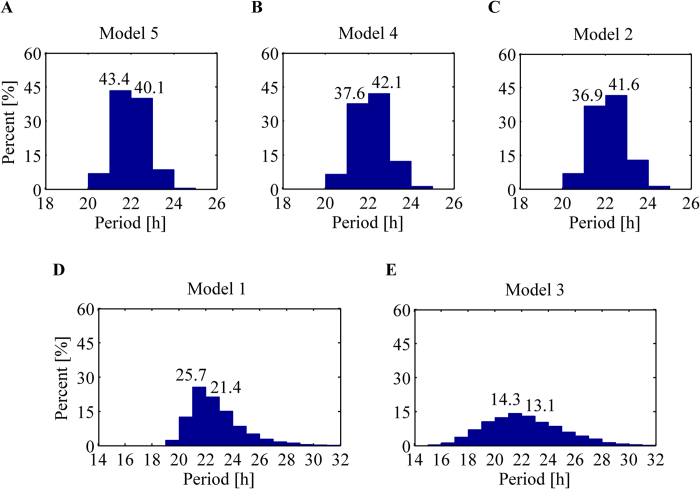
Histograms of period distribution obtained by parameter random perturbations. Top panel displays *more* robust models corresponding to (**A**) a mixed model of a Goodwin oscillator combined with Model 1, (**B**) a Goodwin oscillator, and (**C**) a negative-positive feedback loop with autocatalysis. Bottom panel displays *less* robust models corresponding to (**D**) a substrate-depletion oscillator with a reversible reaction, and (**E**) a negative-positive feedback loop with inhibitory degradation.

Period distributions obtained from random perturbations of each model are illustrated by histograms in Fig. 3. This shows two classes, more robust (top panel) and less robust (bottom panel) models. Three models in the top panel, 5, 4, and 2 in order of insensitivity, are highly robust in the sense that most periods corresponding approximately to 83.5%, 79.7% and 78.5% of all random perturbations lie within 22 ± 1 hours, while the period with default values is ~22 hours in each model. However, Models 1 and 3 show that periods are distributed with a large range and only 47.1% and 27.4% of all perturbations remain within 22 ± 1 hours, respectively.

Additionally, we evaluate the local sensitivity of each parameter for five models as follows:


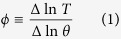


where T represents the period of the sustained oscillatory system and *θ* represents any parameter within the system. This local sensitivity *ϕ* measures approximately the ratio of relative changes near the default parameter values. Table 2 displays the local sensitivity of each parameter with ±20% variations for five models. As highlighted in the table, there are a couple of highly sensitive parameters in Models 1 (*η* and *k*_4_) and 3 (*k*_1_ and *k*_3_), which were verified to be less robust models in the period analysis above. Consistently, the periods of Models 2, 4, and 5 are shown to be less sensitive to the local change of all parameter values, which is in line with the result above. This illustrates that the existence of highly sensitive parameters affects the period robustness of models. Note that if the period of oscillations is a homogeneous function of reaction rate constants of degree −1, the summation theorem states the following relation^55^,^56^:


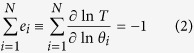


where *N* is the number of parameters for a model. Here 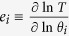 is often called *control coefficients* or *elasticity*. The local sensitivity *ϕ* measures the local change for deviations from the reference values and the sum of the local changes approximately achieves the above relation.

It is also worth pointing out that the shape of solution curves may play an important role in determining the robustness of the system in the context of maintaining the frequency. The numerical solutions of Models 2, 4, and 5, which were classified into more robust models to parameter random perturbations, show approximately sinusoidal curves, whereas those of Models 1 and 3 take the non-sinusoidal shape with radical change.

We searched reasonable parameter spaces where we could vary 40% of all of the parameters in each model by performing extensive one- and two-parameter bifurcation analysis. Therefore, each model has different parameter sets, which may be the main reason for the observed differences in robustness. In order to investigate whether the above hierarchy of robustness is due to parameter space or network topology, we modified the wirings of Models 1, 2, and 4 to resemble the network topologies of Models 2, 3, and 5, respectively, while maintaining the original parameters. Model 1 is modified into Model 1′ by incorporating a negative feedback loop (Supplementary Text S3), Model 2 is modified into Model 2′ by changing the location of positive feedback loop (Supplementary Text S4), and Model 4 is modified into Model 4′ by adding an autocatalytic process (Supplementary Text S5). All of these changes in the corresponding wirings are summarized in Supplementary Table S1. Note that we used the same parameter values in the original model and its modified model except the new parameters for the additional kinetic reaction. Although Model 1 and Model 1′ take the similar shape curves, Model 1′ is more robust in response to random perturbation of parameters. Similarly, Models 4 and 4′ display similar sinusoidal curves in the same parameter space, but Model 4′ with an autocatalysis is less sensitive to random parameter perturbations than Model 4. Furthermore, Model 2′ becomes less robust when the positive feedback is manifested via inhibition of protein degradation, which is similar to Model 3. Our data indicate that modifications in network topology result in different sensitive parameters in the system affecting the robustness of the system in the presence of external noise.

#### Stochastic simulations

In the previous section, we investigate a global sensitivity of the period by simultaneously varying all parameters except for a subset of parameters (i.e., Hill coefficients and critical thresholds), which are assumed to be insensitive to external noise. In this section, we perform stochastic simulations to investigate period sensitivity in the presence of molecular noise. We first develop five stochastic models corresponding to the five ODE models introduced in previous sections, and then investigate the effect of stochasticity on the period of the five model.

To analyze the period sensitivity in stochastic models, we employ the stochastic simulation algorithm developed by Gillespie^27^,^28^, which provides an exact sample path of the Markov chain model. A parameter *N* in Gillespie algorithm is the volume factor that controls the number of molecules of species and thus varies the level of stochastic noise in the system. As an example, Table S5 in the Supplementary Text S6 illustrates the stochastic version of Model 3. In this work, each stochastic simulation was run for 4,000 h and we removed the first 1,000 h to allow for transients to subside. Phase portraits of both deterministic and stochastic models in phase plane demonstrate that for the lower value of *N*, the stochastic trajectories deviate considerably from the limit cycle of the ODEs due to a large fluctuation. As the volume size increases, the fluctuation of the stochastic oscillations decreases, that is, the system becomes more insensitive to molecular noise. Similarly, oscillations of the stochastic system are damped slowly (Supplementary Text S6). We also analyze the frequencies of stochastic trajectories using fast Fourier Transformation (FFT) and compare with those of the systems of ODEs, which indicate that the volume factor controls the number of molecules and thus the amplitude of fluctuations around the deterministic limit cycle depends on the number of molecules in the system (Supplementary Text S6).

**Figure 4:**
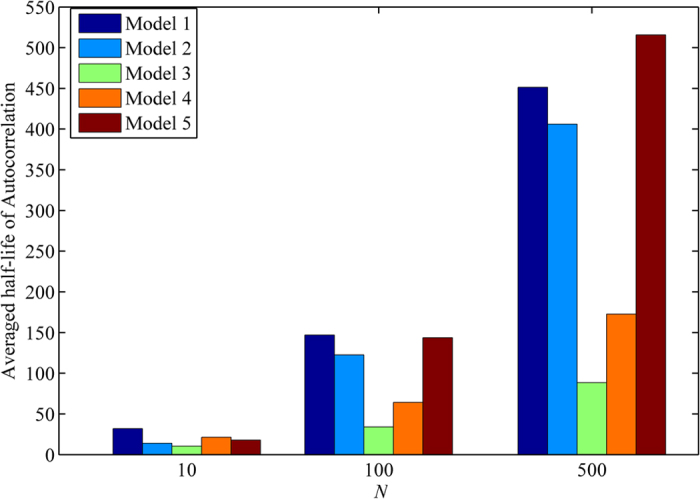
Averaged Half-life of autocorrelations of five models. For each model, the half-life of autocorrelation is measured in each species and then is averaged over species within the model. *N* is varied with 10, 100, and 500.

As known, the half-life of the autocorrelation, the time interval corresponding to 50% decrease from the start, is a useful tool to measure the robustness of periodic systems in the presence of molecular noise^23^,^24^. In Fig. 4, the half-life of autocorrelations is averaged over all species within each model and is compared between five models as *N* varies. Note that the half-life of autocorrelation functions can vary in different species within the model. In the presence of strong noise, corresponding to *N* = 10, the half-life of five models ranks them in the order of insensitivity as Model 1, Model 4, Model 5, Model 2, and Model 3. However, as *N* increases, which reduces the noise strength in the system, the ranking of robust models is switched around. For example, when *N* = 500, the half-life of five models is classified into two groups, more robust (Model 5, Model 1, and Model 2 in order of insensitivity) and less robust (Model 4 and Model 3 in order of insensitivity) models. As we can see, the half-life of Model 5 increases drastically with an increasing volume size, indicating that Model 5 is relatively more robust than other models as the fluctuations in the number of molecules decreases. In contrast, Model 4 becomes less robust as the strength of noise decreases. Interestingly, Model 3 maintains the low half-life steadily, resulting in the most sensitive model to molecular noise. Recall that Model 3 is also the least robust model when the random variations of parameter values were taken into account, see Fig. 3. Model 1 with random parameter perturbations was also classified into a less robust group; however, its stochastic model becomes more robust in the presence of molecular noise.

**Figure 5:**
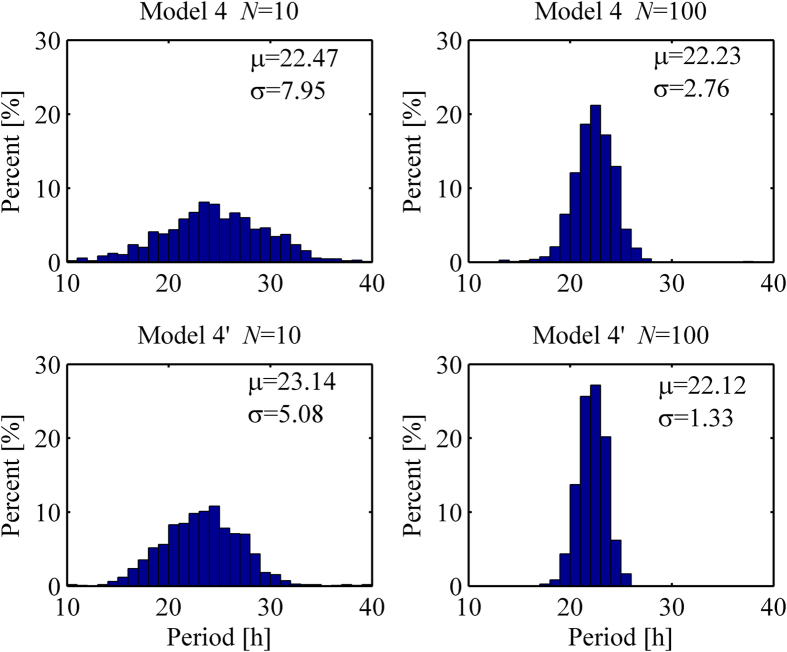
Period distributions for Models 4 and 4′. Model 4 and Model 4′ differ by the existence of autocatalysis, in which Model 4′ includes an autocatalytic process on its protein modification. Each panel is obtained from 1100 consecutive cycles of a stochastic simulation when *N* = 10 and 100. The values ***μ*** and ***σ*** stand for the mean period and standard deviation, respectively.

It is reported that the model becomes more robust to molecular noise in the presence of a positive feedback^26^. Hence we investigate the effect of a positive feedback with stochastic versions of Model 4 and Model 4′, which is distinguished by the absence or presence of autocatalytic process, respectively. Figure 5 displays histograms showing the distribution of periods obtained from 1,100 consecutive cycles of each stochastic simulation with Model 4 (top panel) and Model 4′ (bottom panel) when *N* = 10 and *N* = 100. For each volume size, the histograms are more concentrated in Model 4′, which proves that a positive feedback strengthens the robustness to molecular noise. The two distributions from Models 4 and 4′ are statistically different with p-value less than 0.0001 using Kolmogorov-Smirnov test for each volume size.

**Figure 6:**
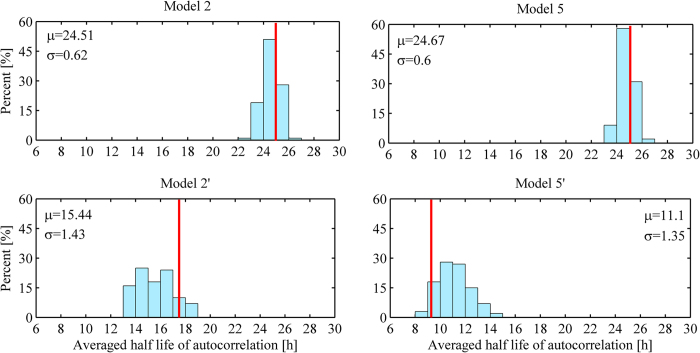
Distribution of averaged half-life of autocorrelations. Model 2 and Model 5 adopt positive regulation via autocatalytic process, while Model 2′ and Model 5′ adopt positive regulation via inhibitory degradation. With each model, we considered 100 random parameter sets with stochastic noise and obtained the distribution of averaged half-life of autocorrelations. Red line in each panel exhibits the averaged half-life of autocorrelation with the default parameter set. For each case, a total of 1000 realizations of the Gillespie algorithm with volume factor *N* = 10 were performed. All parameter values were varied except critical thresholds and Hill coefficients. The values ***μ*** and ***σ*** in each panel stand for the mean and standard deviation of averaged half-life of autocorrelations, respectively.

As mentioned before, we considered two types of positive feedback, i.e., autocatalytic process and inhibitory degradation. We wondered which positive feedback does enhance the robustness of period in the presence of both internal and external noises. To test this, we modified Model 2 and Model 5 by switching autocatalysis to inhibitory degradation, called Model 2′ and Model 5′, respectively (see Supplementary Table S1). We found that positive feedback via autocatalysis makes the system more robust in period regardless of noise type, see Fig. 6. In this figure, we considered 100 random parameter sets together with stochastic noise and obtained the distribution of averaged half-life of autocorrelations. It is clear that the distribution is more concentrated at the higher averaged half-life when the autocatalytic positive regulation is employed in the system. Therefore, we can conclude that the autocatalytic positive feedback enhances the robustness of period in the presence of both external and internal noises when compared with inhibitory degradation model.

The above simulation results demonstrate that oscillatory systems behave differently depending on the type of noises and the strength of molecular noises, suggesting that two different approaches, random perturbations of parameters and stochastic inherent noises, are necessary to investigate the robustness of oscillations in the presence of various noises.

## Conclusions

We have investigated five simple models of biochemical oscillators with different combinations of feedback loops. Biological examples of the above feedback loops are found in biology ranging from substrate-depletion models in glycolytic oscillators to circadian rhythms that utilize both positive and negative feedback loops^3^,^4^. In this paper, we thoroughly examined different dynamical behaviors and robustness of these simple models in the context of period maintenance and variations.

The analysis of these models shows that differences in network topology result in dramatic changes in nonlinear dynamics of the system. In addition, we observe that sensitive parameters that determine the periodicity of the system change depending on the wiring, and demonstrate that Models 2, 4, and 5 are more robust than Models 1 and 3 based on our sensitivity analysis where a set of selected parameters are perturbed randomly. On the other hand, we observe that the robustness of most models is altered when inherent stochasticity is embedded in the system. For example, Model 4 becomes less robust, while Model 5 becomes more robust as the inherent noise is reduced. Model 1 is less robust in the presence of external noise, whereas it becomes more robust in the presence of stochastic noise. In general, Model 3 is always the least robust model regardless of noise type. These simulation results suggest that different network topologies and various types of noises may yield different robustness, which is consistent with our sensitivity and robustness analyses with Models 1–5 and their modified models. Our general results also suggest that oscillatory systems with negative feedback loops such as Models 2, 4, and 5 exhibit more robust maintenance of period in the presence of external noise (Fig. 3) except the model that includes positive feedback via inhibitory degradation in addition to the negative feedback loop (Model 3). On the other hand, positive feedback loops strengthen the period robustness in the presence of internal noise (Fig. 6). This is consistent with a previous report showing that wiring diagrams with negative feedback maintain the period more robust than those with positive feedback in the presence of parameter variations^21^. The dynamical behavior of oscillatory systems may dramatically change depending on parameter space, kinetic equations, and solution curves. Therefore, our future work will involve a more comprehensive global search of parameter space and periodic shape for each network topology.

In this report, we focused on the robustness of oscillatory systems in the context of period. In our future work, we will extend our studies to investigate robustness of the amplitude of oscillations and its relationship with period changes. The age-related reduction of amplitude of synchronized circadian rhythms in the mammalian master clock, suprachiasmatic nucleus (SCN), is suggested to be responsible for decrease of electrical activities in the SCN and locomotor behavior in mice^57^. Moreover, small circadian amplitude of physiological measurements (e.g. heart rate, oral temperature rhythms, etc.) is associated with intolerance to shift work^58^. It is also important to note that the entry into the mitosis is regulated by a threshold level of active CycB/CDC2 complex, and the mitotic entry does not occur if this threshold is not reached^59^. Therefore, it is critical to identify key parameters and network topologies that determine amplitude of oscillatory systems.

Our data will be useful to elucidate underlying molecular mechanisms of biochemical oscillators. For example, one can test period dependencies on several steps of biochemical reactions (e.g. transcription, translation, protein modification, etc.), and infer molecular wiring based on our data. We acknowledge, however, that the analyzed models may be too simple to simulate the detailed nature of complex biological systems. On the other hand, our data will be informative in constructing synthetic circuits for biochemical oscillators. One can design molecular constructs based on the desired outcomes with period-determining sensitive parameters in mind. It will be particularly interesting to experimentally validate wiring-dependent robustness of biochemical oscillators using synthetic biology^60^. Our analyses of these simple models will be a good starting point to analyze or build biochemical oscillators.

## Additional Information

**How to cite this article**: Caicedo-Casso, A. *et al.* Robustness and period sensitivity analysis of minimal models for biochemical oscillators. *Sci. Rep.* **5**, 13161; doi: 10.1038/srep13161 (2015).

## Supplementary Material

Supplementary Information

## Figures and Tables

**Table 1:** Five systems of biochemical oscillators.

Wiring diagram	Systems of ODEs	Numerical solutions
See Model 1 Wiring	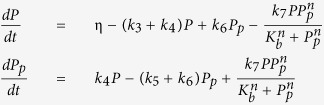	See Model 1 Numerical Solutions
See Model 2 Wiring	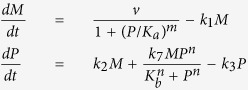	See Model 2 Numerical Solutions
See Model 3 Wiring	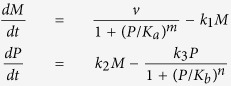	See Model 3 Numerical Solutions
See Model 4 Wiring	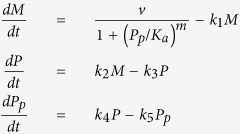	See Model 4 Numerical Solutions
See Model 5 Wiring	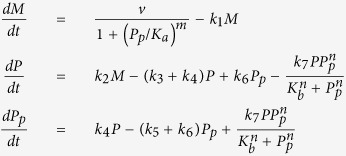	See Model 5 Numerical Solutions

Left, middle, and right columns show molecular wiring diagrams, corresponding systems of ODEs, and their numerical solutions, respectively. Parameter values for each model are given as: (Model 1) *η* = 2.5795, *k*_3_ = 0.01, *k*_4_ = 0.16, *k*_5_ = 0.33, *k*_6_ = 0.21, *k*_7_ = 2.69, *K*_*b*_ = 10, *n* = 8, (Model 2) *v* = 3.26, *k*_1_ = 0.045, *k*_2_ = 0.161, *k*_3_ = 0.869, *k*_7_ = 2.174, *K*_*a*_ = 5.5, *K*_*b*_ = 15, *m* = 3, *n* = 2, (Model 3) *v* = 148, *k*_1_ = 0.207, *k*_2_ = 0.741, *k*_3_ = 2.561, *K*_*a*_ = 1.1, *K*_*b*_ = 3, *m* = 3, *n* = 2, (Model 4) *v* = 18.18, *k*_1_ = 0.182, *k*_2_= 2.02, *k*_3_ = 0.172, *k*_4_ = 0.141, *k*_5_ = 0.182, *K*_*a*_ = 5, *m* = 10, and (Model 5) *v* = 24.44, *k*_1_ = 0.236, *k*_2_ = 2.356, *k*_3_ = 0.059, *k*_4_ = 0.134, *k*_5_ = 0.142, *k*_6_ = 0.063, *k*_7_ = 0.629, *K*_*a*_ = 3, *K*_*b*_ = 10, *m* = 8, *n* = 4.

**Table 2:** Local sensitivity of parameters for five models.

Model 1		Model 2		Model 3		Model 4		Model 5	
parameter	*ϕ*	parameter	*ϕ*	parameter	*ϕ*	parameter	*ϕ*	parameter	*ϕ*
_*η*_	**−1.7**	*v*	−0.302	*v*	0.07	*v*	0.007	*v*	−0.01
*k*_3_	0.07	*k*_1_	−0.264	*k*_1_	**−1.994**	*k*_1_	−0.342	*k*_1_	−0.338
*k*_4_	**−0.599**	*k*_2_	−0.397	*k*_2_	0.072	*k*_2_	0.007	*k*_2_	−0.011
*k*_5_	0.198	*k*_3_	−0.109	*k*_3_	**0.893**	*k*_3_	−0.313	*k*_3_	−0.024
*k*_6_	0.169	*k*_7_	0.09			*k*_4_	0.007	*k*_4_	−0.407
*k*_7_	0.136					*k*_5_	−0.343	*k*_5_	−0.229
								*k*_6_	0.017
								*k*_7_	−0.033
