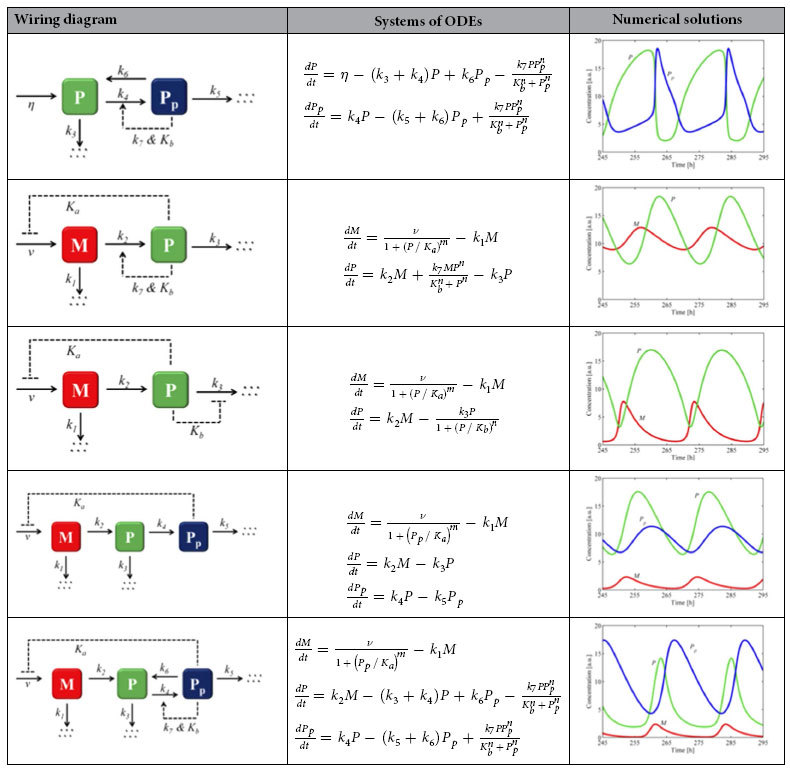# Corrigendum: Robustness and period sensitivity analysis of minimal models for biochemical oscillators

**DOI:** 10.1038/srep18504

**Published:** 2016-01-12

**Authors:** Angélica Caicedo-Casso, Hye-Won Kang, Sookkyung Lim, Christian I. Hong

Scientific Reports 5: Article number: 1316110.1038/srep13161; published online 08122015; updated on 01122016

In this Article, the images depicting the Wiring Diagarms and Numerical solutions for models 1 to 5 have been omitted. The correct Table 1 appears below.

In Table S5, the transition values for Reaction 1 *′M* → *M* + *1′* and Reaction 2 *′M* → *M* − *1′* were incorrectly given as *′M* → *M *− *1′* and *′M* → *M* + *1′* respectively.

## Figures and Tables

**Table 1:**