# Supplementary material for: Robustness and period sensitivity analysis of minimal models for biochemical oscillators
Source: Sci Rep. 2015 Aug 12;5:13161. doi: 10.1038/srep13161 (PMC4542697; doi:10.1038/srep13161)
Supplement: Supplementary Information [file srep13161-s1.pdf]

Supplementary Information

Robustness and period sensitivity analysis of minimal models for biochemical oscillators

Angélica Caicedo-Casso[1,2], Hye-Won Kang[3], Sookkyung Lim[1*], and Christian I. Hong[4*]

1. Department of Mathematical Sciences, University of Cincinnati, Cincinnati, OH 45221, USA
2. Departamento de Matemáticas, Universidad del Valle, Cali, Valle, COL
3. Department of Mathematics and Statistics, University of Maryland at Baltimore County, Baltimore, MD 21250, USA
4. Department of Molecular and Cellular Physiology, University of Cincinnati, Cincinnati, OH 45267, USA

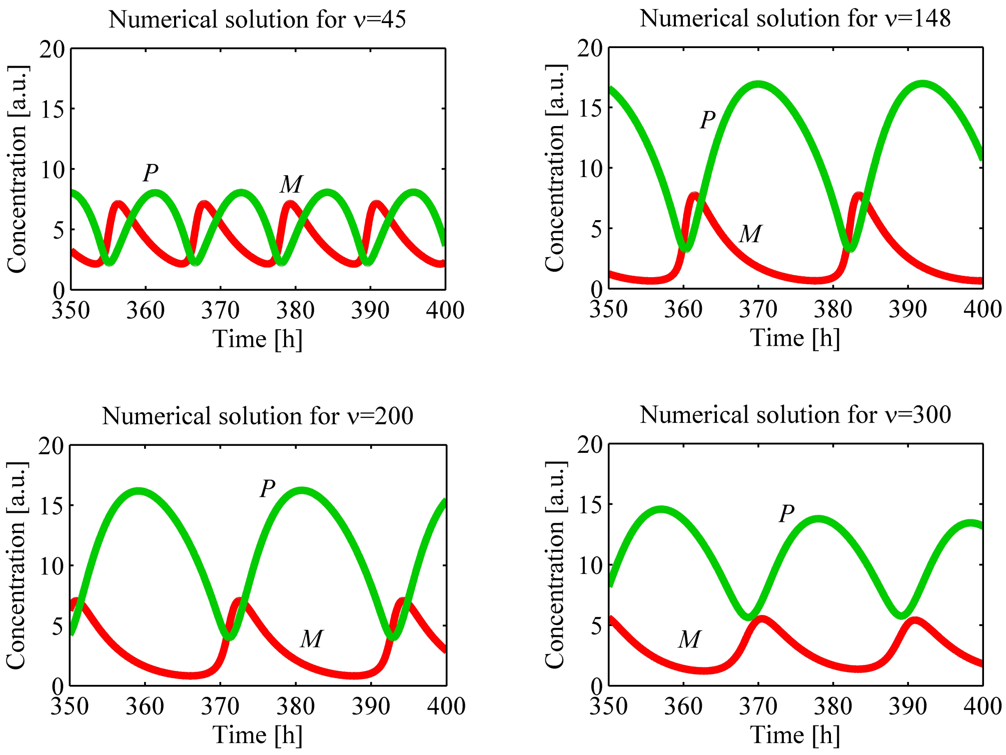

Figure S1. Numerical solutions at different values of ν in Model 3. This figure shows numerical solutions of Model 3 when ν is given as 45, 148, 200, and 300. The period of Model 3 initially increases as ν increases and then the period reaches its maximum at $\nu = 148$. However, the period decreases with increasing ν when $\nu > 148$, in which prolonged protein abundance actually decreases, resulting in a shorter duration of negative feedback.

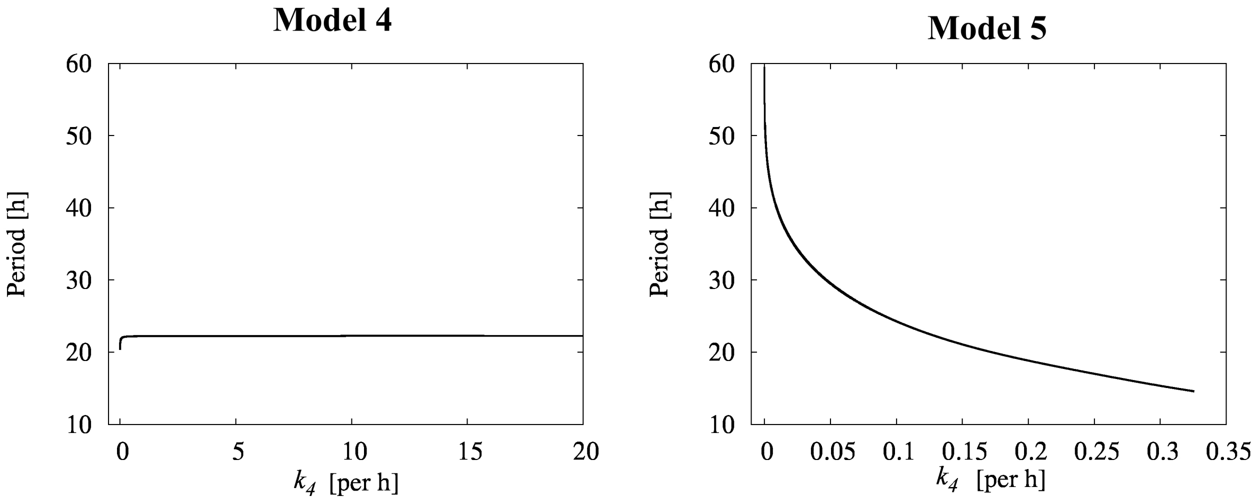

Figure S2. Period as a function of the synthesis rate of phosphorylated protein k_4. Parameter values are described in Table 1 in the main text. Whereas the period remains steady in Model 4, the period decreases rapidly as k_4 increases in Model 5. Model 5 demonstrates that for low values of k_4, the system takes a while to reach the threshold (K_b) for the autocatalytic process to occur. The reversible reaction of protein modification (e.g. dephosphorylation) further delays accumulation of phosphorylated protein. As k_4 increases, the threshold for autocatalysis is achieved faster, and the subsequent rapid increase of P_p leads to faster inhibition of the transcription of mRNA.

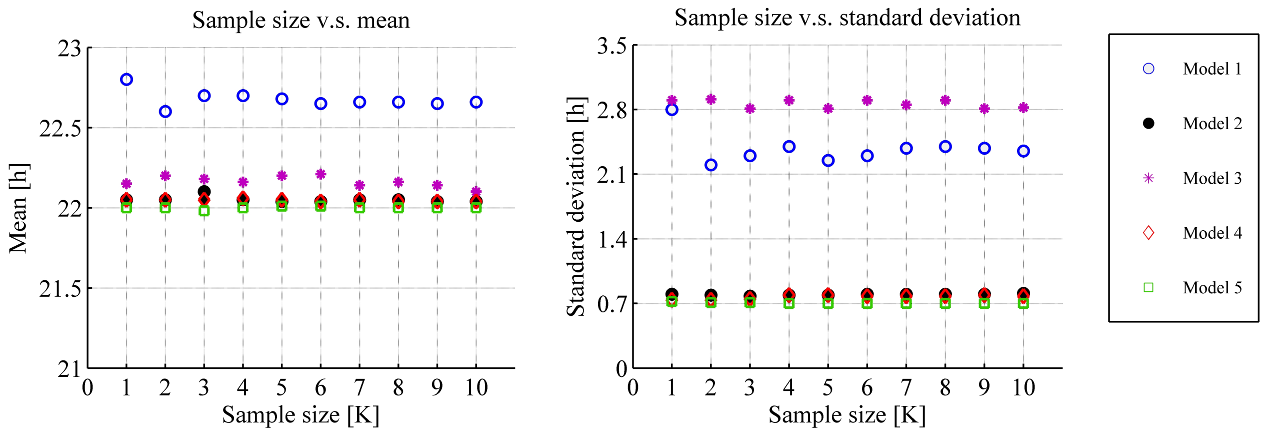

Figure S3. Mean and standard deviation of periods as a function of sample size. For each model, mean and standard deviation of period distribution are measured as the sample size varies from 1,000 to 10,000. Each random perturbation of parameter values lies within ±20% change from each default parameter value.

Table S1. Modified models

<table>
<tr><th>Wiring diagram</th><th>Systems of ODEs</th><th>Numerical solutions</th></tr>
<tr>
<td>

Model 1'

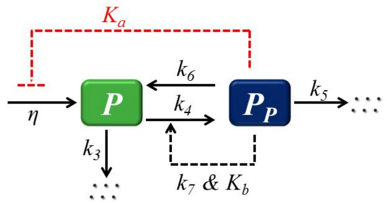

</td>
<td>

$$\begin{aligned}\frac{dP}{dt} &= \frac{\eta}{1+(P_p/K_a)^m} - (k_3+k_4)P + k_6P_p \\ &\quad - \frac{k_7PP_p^n}{K_b^n+P_p^n}, \\ \frac{dP_p}{dt} &= k_4P - (k_5+k_6)P_p + \frac{k_7PP_p^n}{K_b^n+P_p^n}.\end{aligned}$$

</td>
<td>

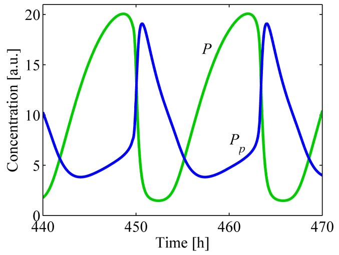

</td>
</tr>
<tr>
<td>

Model 2'

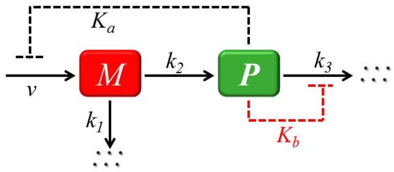

</td>
<td>

$$\frac{dM}{dt} = \frac{\nu}{1+(P/K_a)^m} - k_1M$$

$$\frac{dP}{dt} = k_2M - \frac{k_3P}{1+(P/K_b)^n}$$

</td>
<td>

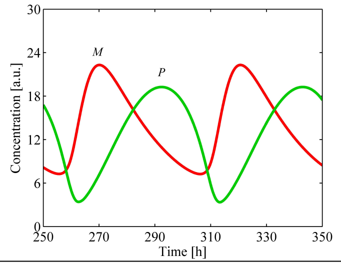

</td>
</tr>
<tr>
<td>

Model 4'

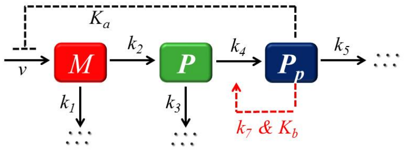

</td>
<td>

$$\begin{aligned}\frac{dM}{dt} &= \frac{\nu}{1+(P_p/K_a)^m} - k_1M, \\ \frac{dP}{dt} &= k_2M - k_3P - \frac{k_7PP_p^n}{K_b^n+P_p^n}, \\ \frac{dP_p}{dt} &= k_4P - k_5P_p + \frac{k_7PP_p^n}{K_b^n+P_p^n}.\end{aligned}$$

</td>
<td>

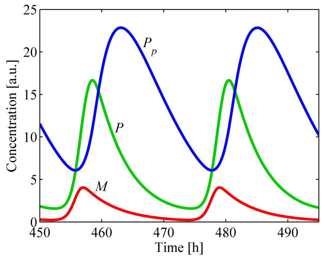

</td>
</tr>
<tr>
<td>

Model 5'

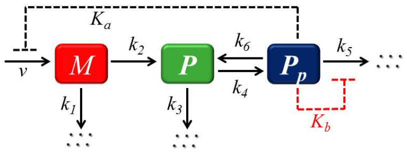

</td>
<td>

$$\begin{aligned}\frac{dM}{dt} &= \frac{\nu}{1+(P_p/K_a)^m} - k_1M, \\ \frac{dP}{dt} &= k_2M - k_3P - k_4P + k_6P_p, \\ \frac{dP_p}{dt} &= k_4P - k_6P_p - \frac{k_5P_p}{1+(P_p/K_b)^n}.\end{aligned}$$

</td>
<td>

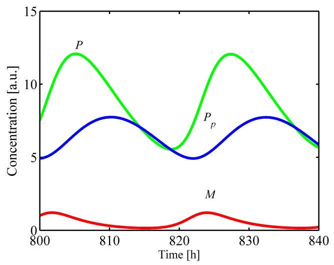

</td>
</tr>
</table>

Left, middle, and right columns show molecular wiring diagrams, corresponding system of ODEs, and numerical solutions, respectively. Parameter values for each model are given as: (Model 1') $\nu = 6, k_3 = 0.01, k_4 = 0.16, k_5 = 0.33, k_6 = 0.21, k_7 = 2.69, K_a = 6, K_b = 10, m = 2, n = 8$. (Model 2') $\nu = 3.26, k_1 = 0.045, k_2 = 0.161, k_3 = 2, K_a = 5.5, K_b = 5, m = 3, n = 2$.(Model 4') $\nu = 18.18, k_1 = 0.182, k_2 = 2.02, k_3 = 0.172, k_4 = 0.141, k_5 = 0.182, k_7 = 0.3913, K_a = 5, K_b = 10, m = 10, n = 4$. (Model 5') $\nu = 24.44, k_1 = 0.236, k_2 = 2.356, k_3 = 0.059, k_4 = 0.134, k_5 = 0.142, k_6 = 0.063, K_a = 3, K_b = 10, m = 8, n = 4$.

Text S1. One-parameter bifurcation analysis for five models

We performed one-parameter bifurcation analysis for five models and present here period diagrams of each model. Each panel in each figure below exhibits the period of oscillations as a function of each parameter, while the other parameter values are held fixed. This analysis was done via computer programs XPP-AUTO and MATLAB.

Model 1: A reversible substrate-depletion oscillator.

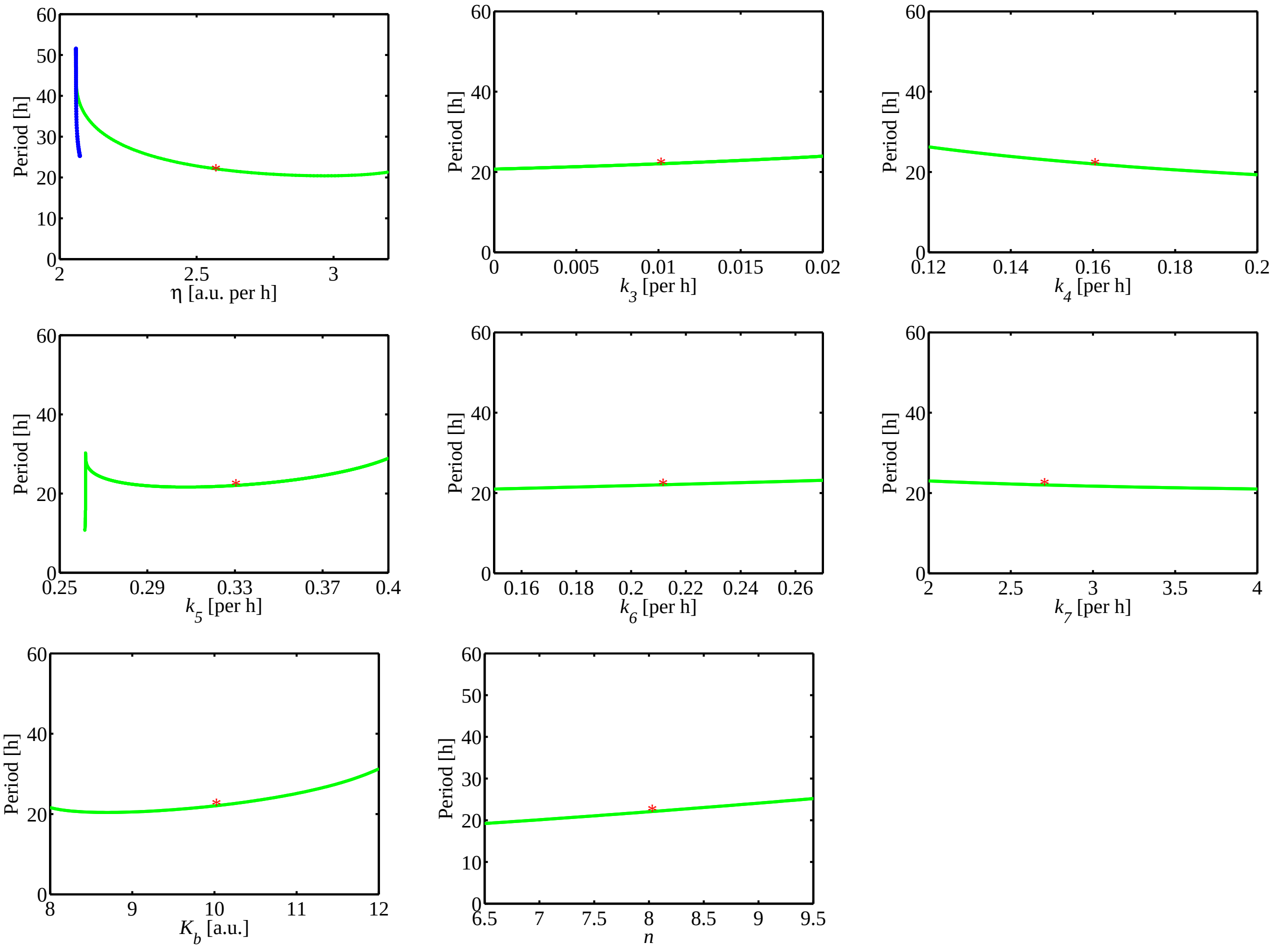

Figure S4. Period diagrams for Model 1. Green and blue curves exhibit stable and unstable periodic orbits, respectively. Red star indicates the default value of each parameter.

Model 2: A negative and positive feedback loop via autocatalysis.

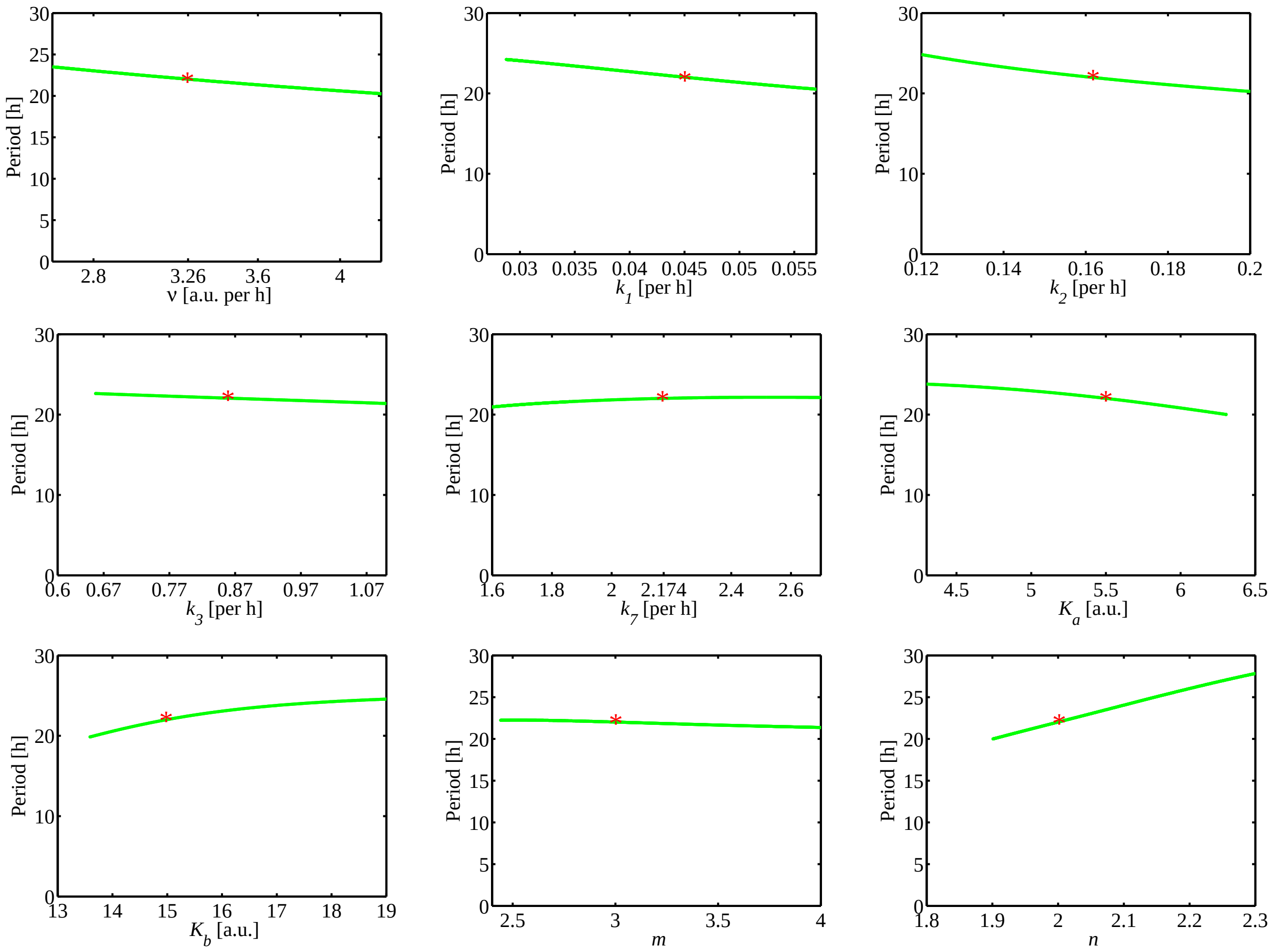

Figure S5. Period diagrams for Model 2. Green and blue curves exhibit stable and unstable periodic orbits, respectively. Red star indicates the default value of each parameter.

Model 3: A negative and positive feedback loop via inhibition of degradation.

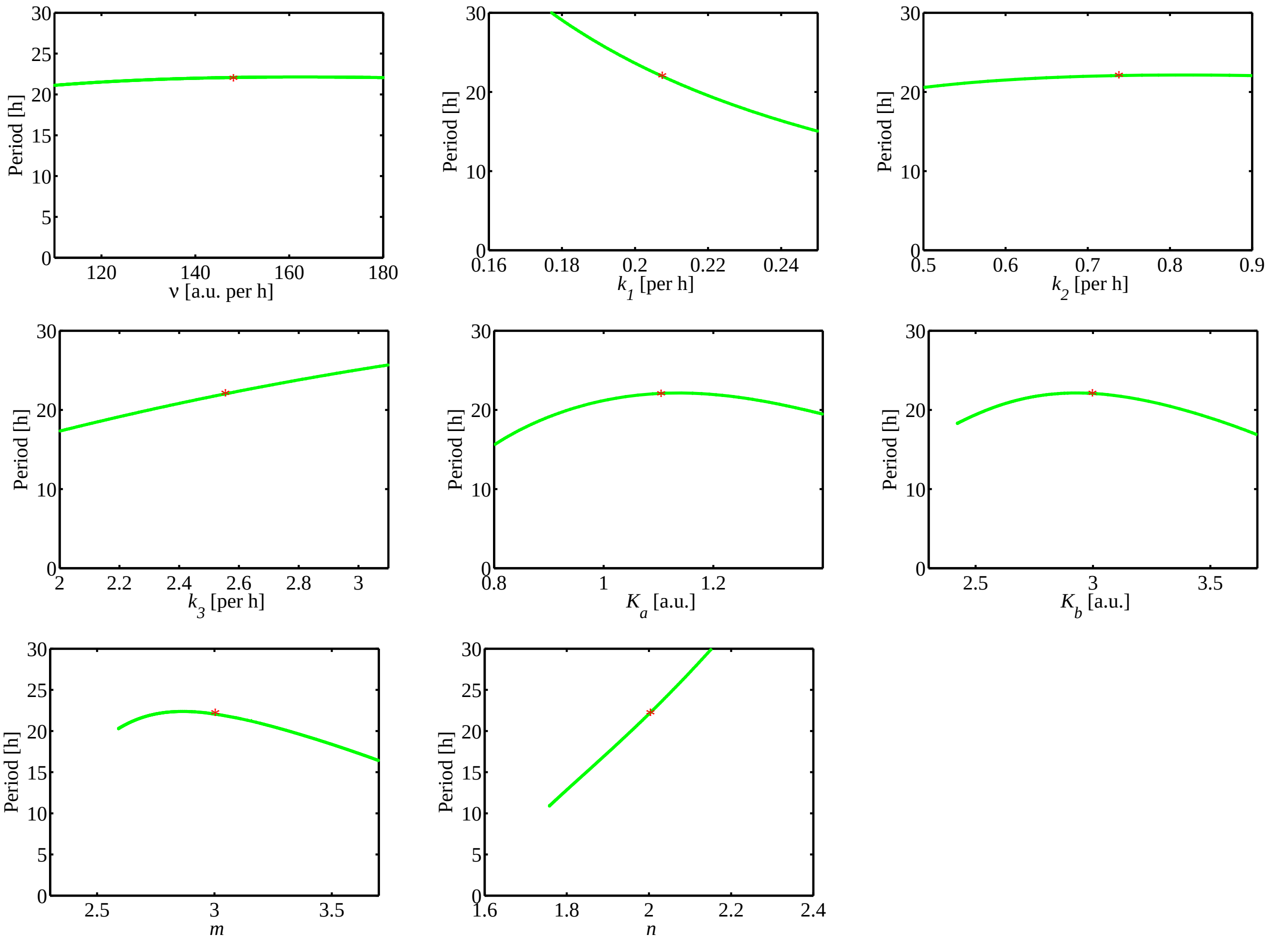

Figure S6. Period diagrams for Model 3. Green and blue curves exhibit stable and unstable periodic orbits, respectively. Red star indicates the default value of each parameter.

Model 4: Goodwin oscillator.

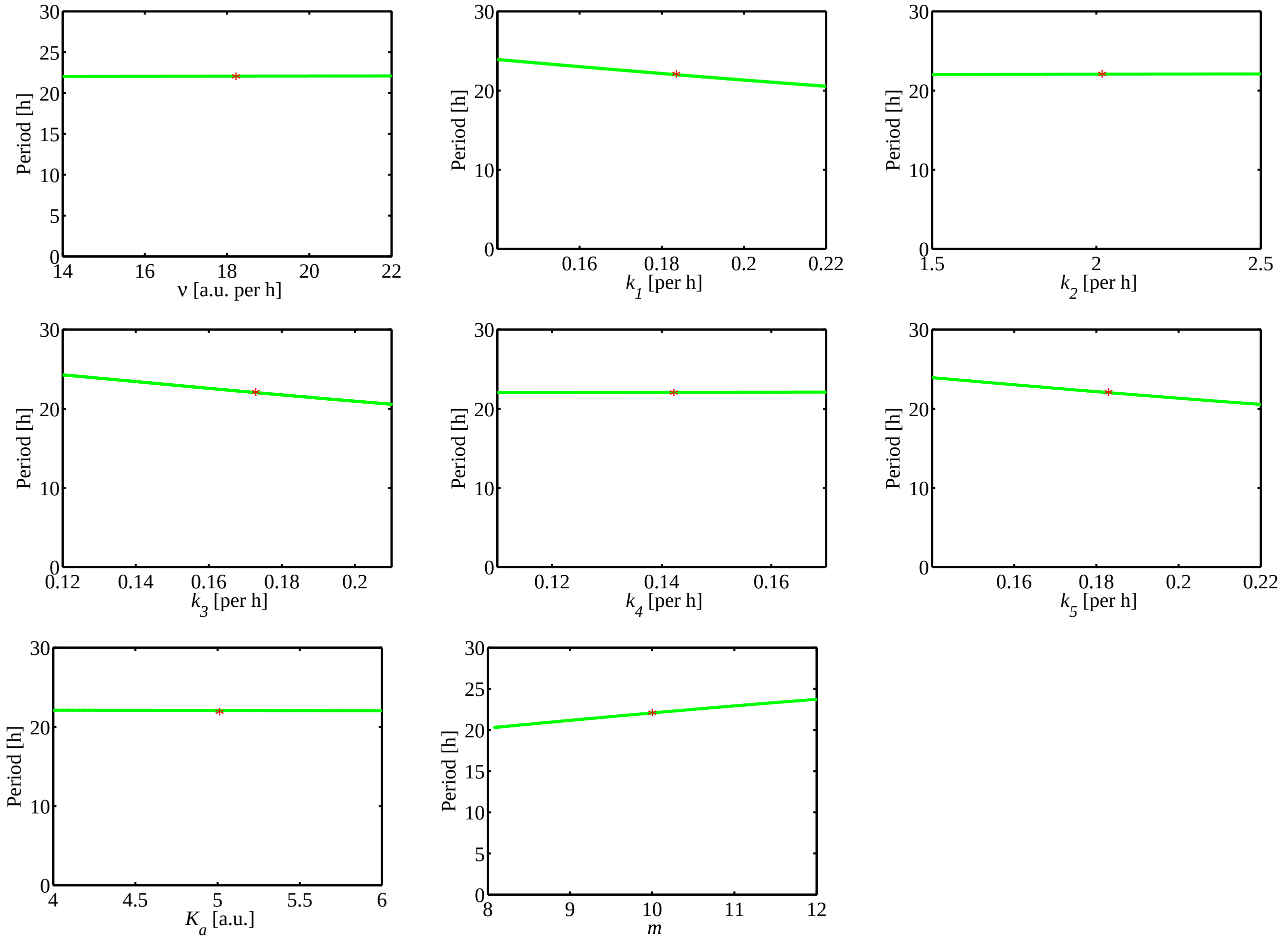

Figure S7. Period diagrams for Model 4. Green and blue curves exhibit stable and unstable periodic orbits, respectively. Red star indicates the default value of each parameter.

Model 5: A modified Goodwin oscillator that incorporates an additional positive feedback loop.

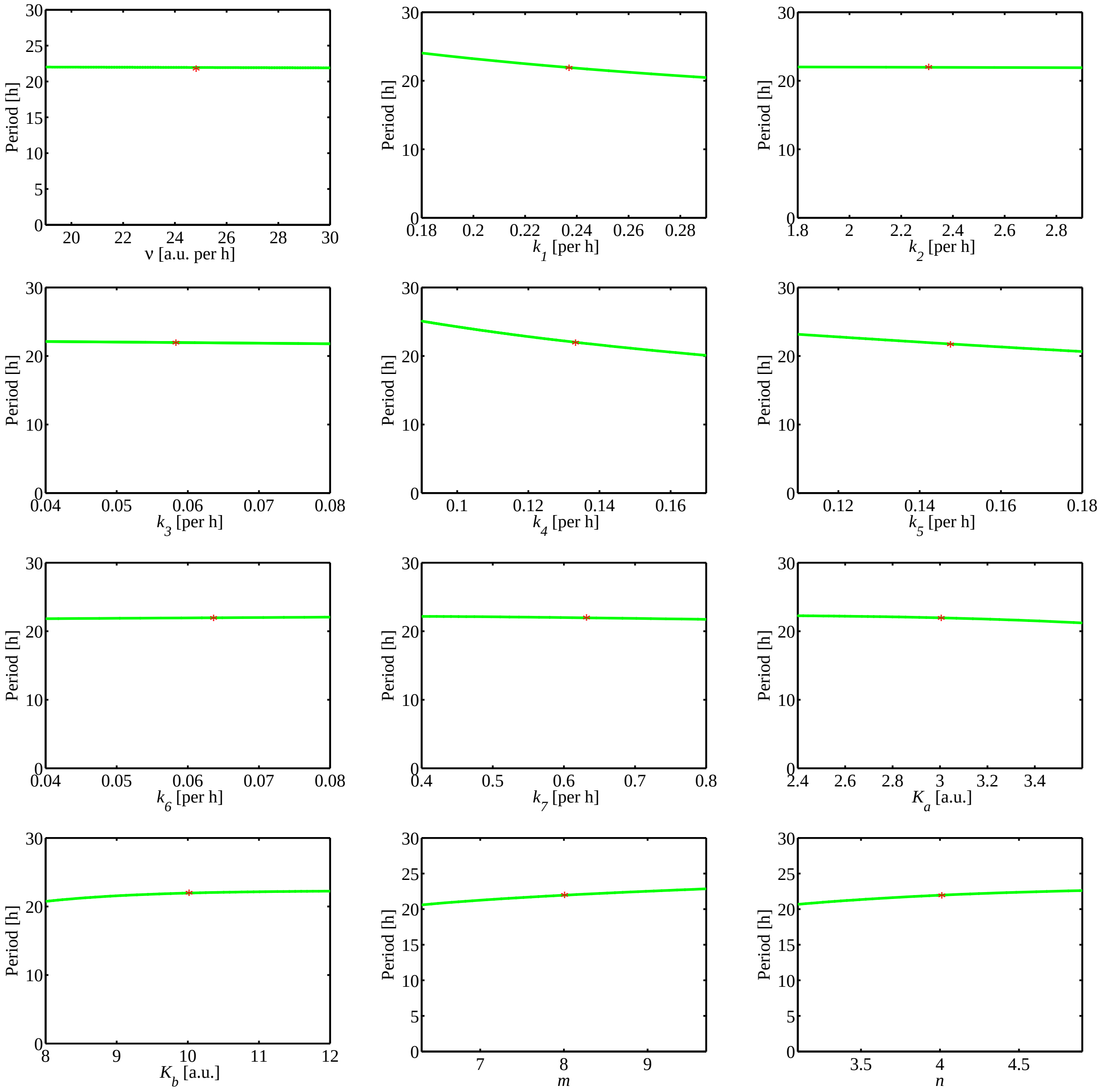

Figure S8. Period diagrams for Model 5. Green and blue curves exhibit stable and unstable periodic orbits, respectively. Red star indicates the default value of each parameter.

Text S2. Phase analysis of Model 1

Figures S9a shows phase portraits of Model 1 with three values of η, which displays P- and P_p-nullclines, and the stable limit cycle solution (solid red curves) obtained from the numerical integration of the ODE system (Table 1 in the main text). Corresponding numerical solutions of Figure S9a are shown in Figure S9b. Similarly, phase portraits of Model 1 with three values of k_5 are described in Figure S9c, and their corresponding numerical solutions shown in Figure S9d.

η is the rate of synthesis of protein (P) and one of the sensitive parameters. k_5 is the degradation rate of phosphorylated protein (P_p) and one of the non-sensitive parameters. We considered 20% variations from the default parameters (η=2.5795, k_5=0.33) to describe the changes of phase diagrams. Increasing η and k_5 moves the P- and P_p-nullclines, respectively, which subsequently changes the location of the fixed point. Phase diagrams demonstrate that the period of the Model 1 is determined by the location of the unstable steady state and corresponding shapes of nullclines, which determines the trajectory of limit cycle. Longer periods are observed when the trajectory of limit cycle spends considerable amount of time close to P_p-nullcline (slow process until the autocatalytic positive feedback kicks in). In any case, we do not observe any drastic differences of phase diagrams between sensitive and non-sensitive parameters.

Bifurcation analysis allows us to study the effect of parameter changes on the behavior of dynamical systems. As examples, Figures S9e-f show bifurcation diagrams as a function of the rate of synthesis (η) and the degradation rate of phosphorylated protein (k_5), respectively. H_1 and H_2 indicate supercritical and subcritical Hopf bifurcation points, respectively. A cyclic fold (saddle-node of limit cycle) occurs at the value of η = 2.06 and k_5 = 0.4184, in which stable and unstable limit cycles coalesce, respectively. Subcritical Hopf bifurcation points are important because they provide insight into where sudden transitions between stable steady states and stable periodic solutions might occur. For more detailed information and other applications, see [1,2].

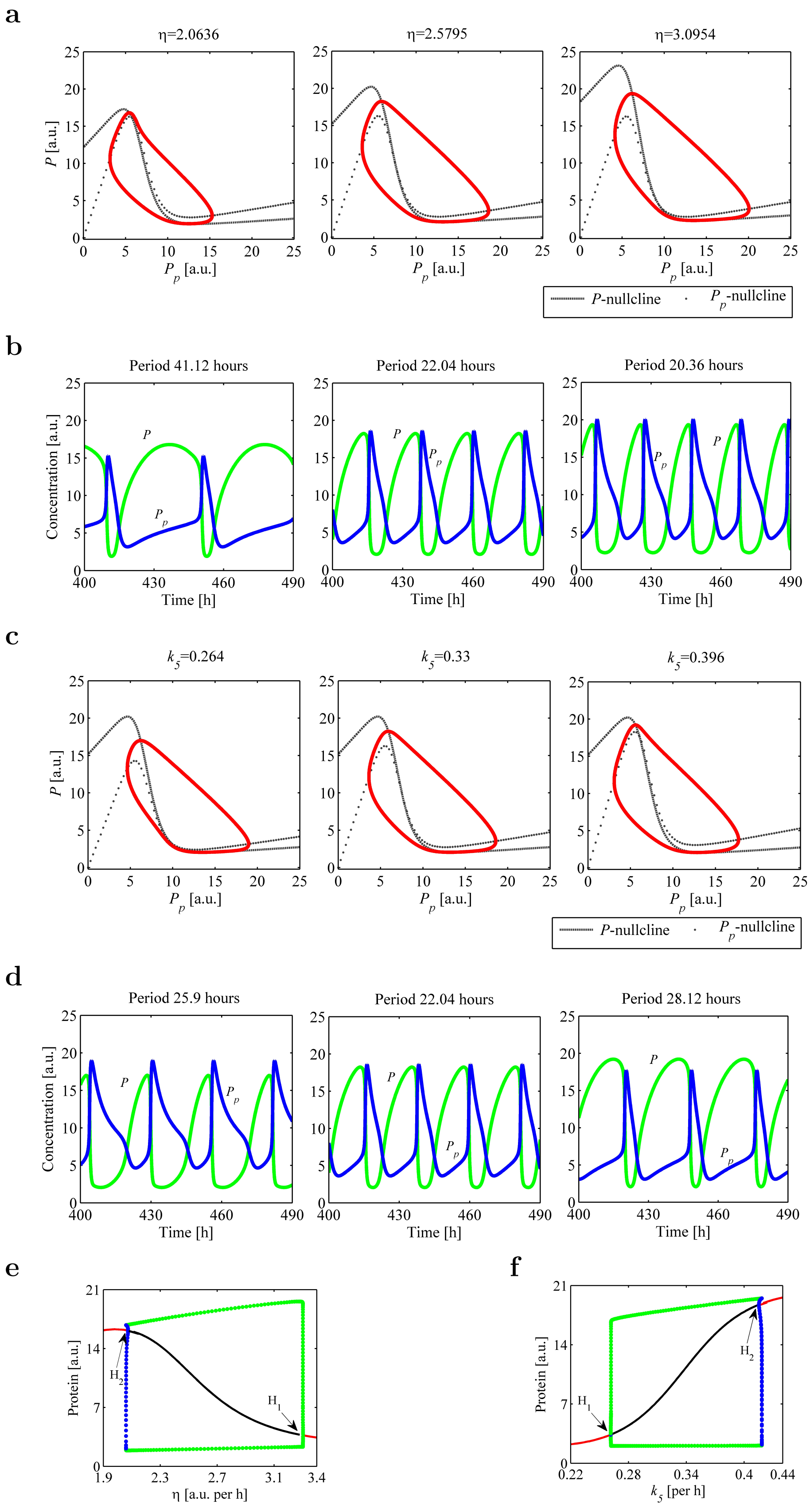

a
η=2.0636
η=2.5795
η=3.0954
P [a.u.]
P_p [a.u.]
P-nullcline
P_p-nullcline
b
Period 41.12 hours
Period 22.04 hours
Period 20.36 hours
Concentration [a.u.]
Time [h]
P
P_p
c
k_5=0.264
k_5=0.33
k_5=0.396
P [a.u.]
P_p [a.u.]
P-nullcline
P_p-nullcline
d
Period 25.9 hours
Period 22.04 hours
Period 28.12 hours
Concentration [a.u.]
Time [h]
P
P_p
e
Protein [a.u.]
η [a.u. per h]
H_1
H_2
f
Protein [a.u.]
k_5 [per h]
H_1
H_2

Figure S9. Limit cycle solutions and bifurcation diagrams for a sensitive and a non-sensitive parameter in Model 1. (a) Phase plane portraits for three different values of η, which is a sensitive parameter. Left, middle, and right panel correspond to -20% of default value, default value, and +20% of default value. P and P_p start with some initial conditions and then reach the stable limit cycle represented by solid red curve. (b) Numerical solutions corresponding to phase portraits in (a). (c) Phase plane portraits for three different values of k_5, which is a non-sensitive parameter. Left, middle, and right panel correspond to -20% of default value, default value, and +20% of default value. (d) Numerical solutions corresponding to phase portraits in (c). (e-f) Display bifurcation diagrams of protein P as η and k_5 vary. Solid red curves, which represent the stable steady state, are connected by a solid black curve in the center which represent the unstable steady state. Filled green and blue circles indicate stable and unstable limit cycles, respectively. H_1 is a supercritical Hopf bifurcation point and H_2 is a subcritical Hopf bifurcation point. Parameter values of η corresponding to these points are $\eta = 3.303$ and $\eta = 2.074$, respectively. Parameter values of k_5 corresponding to the points H_1 and H_2 are given by $k_5 = 0.2615$, and $k_5 = 0.4153$. For $0.206 < \eta < 0.2074$ and $0.4153 < k_5 < 0.4184$, the system exhibits bistability, a stable limit cycle and a stable steady state coexist. Note that the nature of the subcritical Hopf bifurcation point is the existence of unstable limit cycles (empty circles). Parameter values are taken from Table 1 in the main text.

Text S3. A reversible substrate-depletion oscillator with a negative feedback loop (Model 1')

We extended Model 1 to incorporate a negative feedback loop on the protein synthesis (Model 1') and demonstrated that a reversible reaction enlarges oscillatory domain in most parameter space, see Table S2 and Figure S10 below. We used the same parameter values as in Model 1 and introduced new parameters that are related to the negative feedback loop.

Figure S11 shows a histogram of period distribution from 4000 random perturbations of parameter values in Model 1'.

Table S2. A reversible substrate-depletion oscillator with a negative feedback loop.

Wiring diagram	Systems of ODEs	Numerical solution
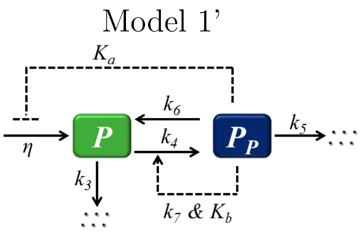	$\frac{dP}{dt} = \frac{\eta}{1+(P_p/K_a)^m} - (k_3+k_4)P + k_6 P_p - \frac{k_7 P P_p^n}{K_b^n + P_p^n}$<br>$\frac{dP_p}{dt} = k_4 P - (k_5+k_6)P_p + \frac{k_7 P P_p^n}{K_b^n + P_p^n}$	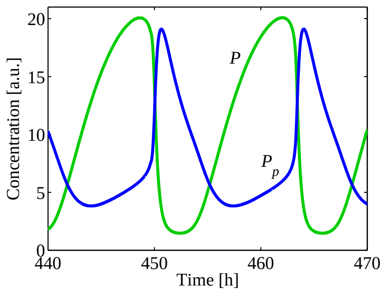

Left, middle, and right columns show a molecular wiring diagram, the corresponding system of ODEs, and the numerical solution, respectively. Parameter values are given as: (Model 1') $\eta = 6$, $k_3 = 0.01$, $k_4 = 0.16$, $k_5 = 0.33$, $k_6 = 0.21$, $k_7 = 2.69$, $K_a = 6$, $K_b = 10$, $m = 2$, $n = 8$. The period of Model 1' is approximately 13.4h.

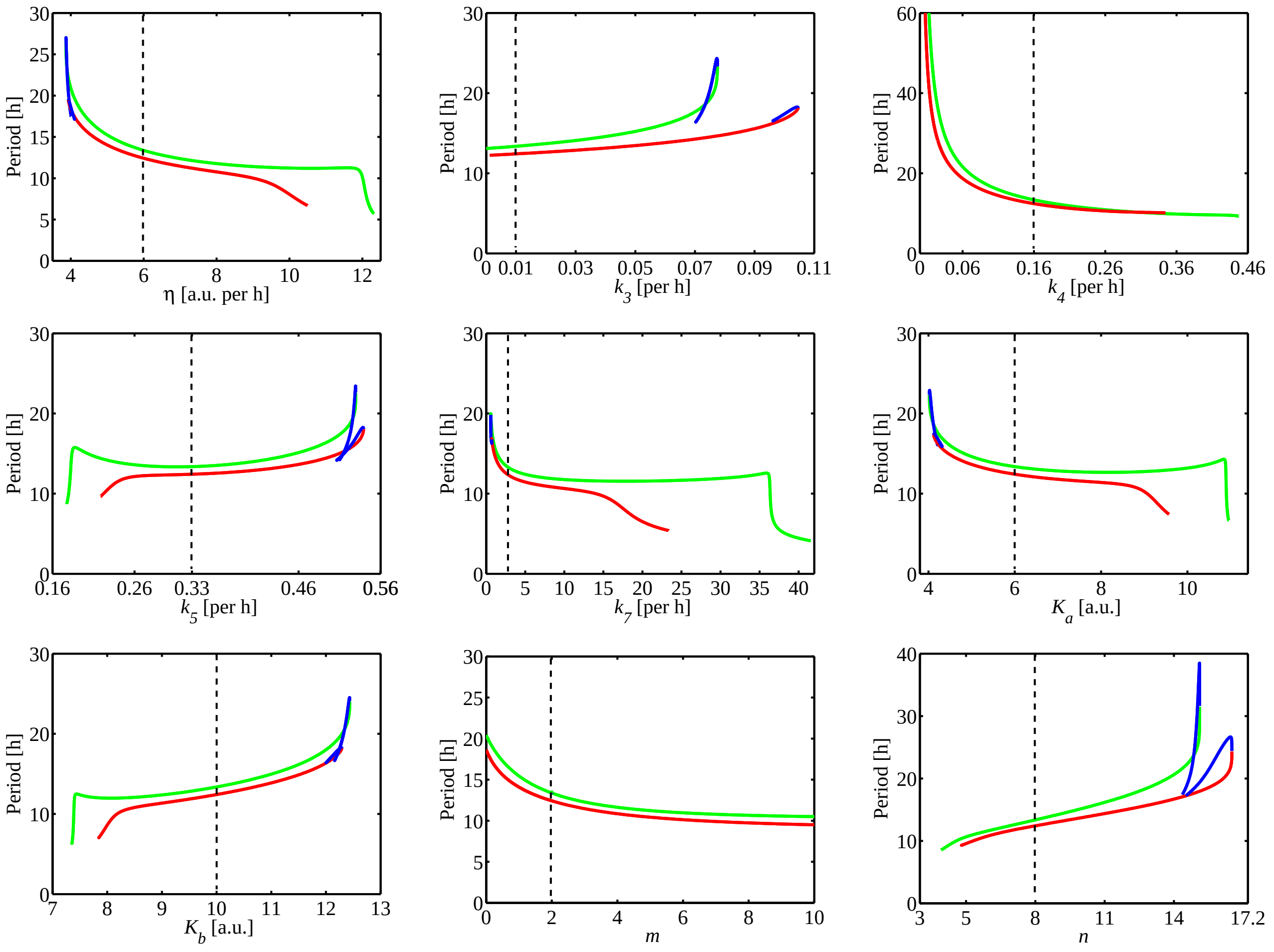

Figure S10. Period diagrams for Model 1' with and without a reversible reaction. Green and red curves indicate stable periodic orbits for Model 1' with ($k_6 = 0.21$) and without ($k_6 = 0$) a reversible reaction, respectively. Blue curve indicates unstable periodic orbits. Dashed line in each panel exhibits the default value of each parameter.

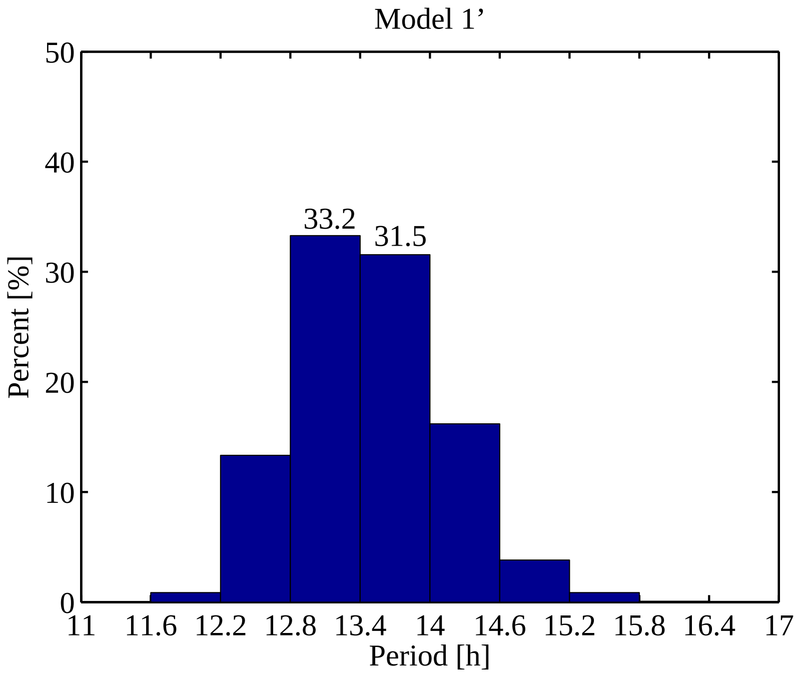

Figure S11. Histogram of period distribution obtained from 4000 random perturbations of parameter values in Model 1'. The period domain is divided every 0.6 hours which is equivalent to 4.55% of 13.4 hours. Note that 4.55% is approximately 1 hour change for histograms of about 22 hours of five periodic models.

Text S4. A modification of Model 2 with positive feedback loop via inhibitory degradation (Model 2')

We changed the location of a positive feedback loop of Model 2 (Model 2') which resembles the network topology of Model 3, and investigated the dependence of robustness on network topology. See Table S3 below for Model 2'. We used the same parameter values as in Model 2 and introduced new parameters that are related to the new location of the positive feedback loop. Our simulation results demonstrate that Model 2' is less robust than Model 2, see Figure S12 below.

Table S3. New location of positive feedback in Model 2.

Wiring diagram	Systems of ODEs	Numerical solution
Model 2' 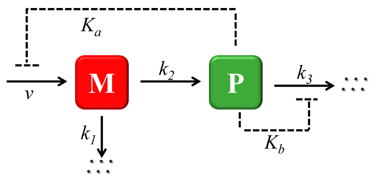	$\frac{dM}{dt} = \frac{\nu}{1+(P/K_a)^m} - k_1 M$<br>$\frac{dP}{dt} = k_2 M - \frac{k_3 P}{1+(P/K_b)^n}$	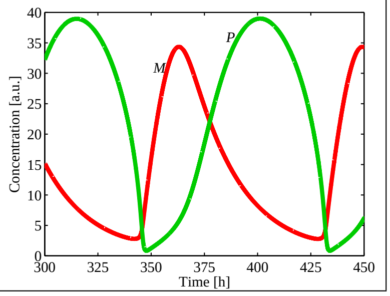

Left, middle, and right columns show a molecular wiring diagram, the corresponding system of ODEs, and the numerical solution, respectively. Parameter values are given as: (Model 2') $\nu = 3.26$, $k_1 = 0.045$, $k_2 = 0.161$, $k_3 = 2$, $K_a = 5.5$, $K_b = 5$, $m = 3$, $n = 2$. The period of Model 2' is approximately 86h.

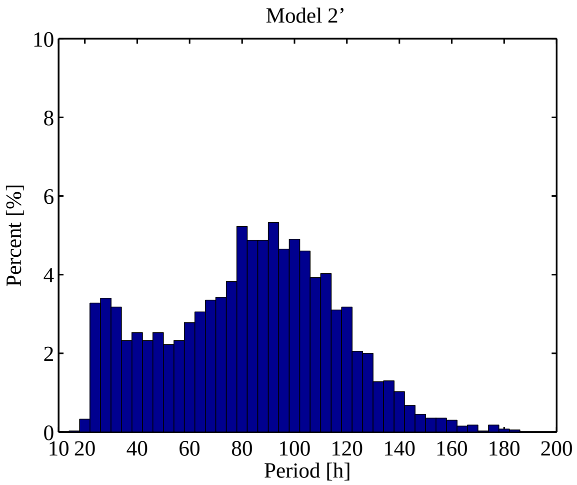

Figure S12. Histogram of period distribution obtained from 4000 random perturbations of parameter values in Model 2'. The period domain is divided every 4 hours which is equivalent to 4.55% of 86 hours. Note that 4.55% is approximately 1 hour change for histograms of ~ 22 hours of five periodic models.

Text S5. A Goodwin model with an irreversible autocatalytic process (Model 4')

We modify the Goodwin model (Model 4) by incorporating an irreversible autocatalytic process on the synthesis of the end product (Model 4'), and investigate how this additional positive feedback mechanism changes the dynamics of the system compared to the Goodwin model. The extended system of ODEs is given in Table S4 below. Parameter values of Model 4' are adopted from the Goodwin model except the parameter values for the autocatalytic process k_7, K_b, and n. Our numerical simulations suggest that an autocatalytic reaction improves the robustness of period for most parameters as previously indicated [6], see Figures S13a - S13c below. However, there is a disadvantage in the inclusion of this autocatalysis. The synthesis rate of end product, k_4, which is one of the insensitive parameters in the Goodwin model, becomes a sensitive parameter in Model 4' (see Figure S13d). This is expected because an autocatalytic process provides a nonlinear switch that will change the period of the system. The period decreases as a function of k_4, because this results in reaching the threshold for autocatalysis faster, which triggers faster inhibition on M.

Table S4. A Goodwin oscillator with autocatalysis.

Wiring diagram	Systems of ODEs	Numerical solutions
Model 4'<br>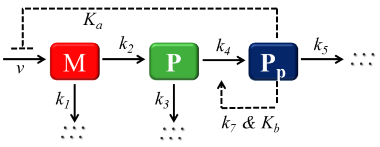	$\frac{dM}{dt} = \frac{\nu}{1+(P_p/K_a)^m} - k_1 M$<br>$\frac{dP}{dt} = k_2 M - k_3 P - \frac{k_7 P P_p^n}{K_b^n + P_p^n}$<br>$\frac{dP_p}{dt} = k_4 P - k_5 P_p + \frac{k_7 P P_p^n}{K_b^n + P_p^n}$	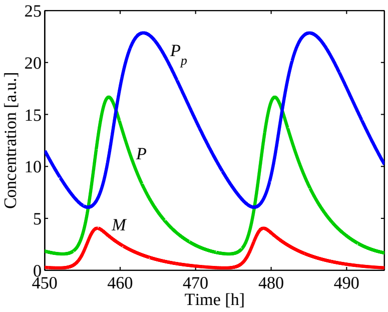

Left, middle, and right columns show a molecular wiring diagram, the corresponding system of ODEs, and the numerical solution, respectively. Parameter values are given as: $\nu = 18.18$, $k_1 = 0.182$, $k_2 = 2.02$, $k_3 = 0.172$, $k_4 = 0.141$, $k_5 = 0.182$, $K_a = 5$, $m = 10$, $k_7 = 0.3913$, $K_b = 10$, $n = 4$. The period of Model 4' is approximately 22 h.

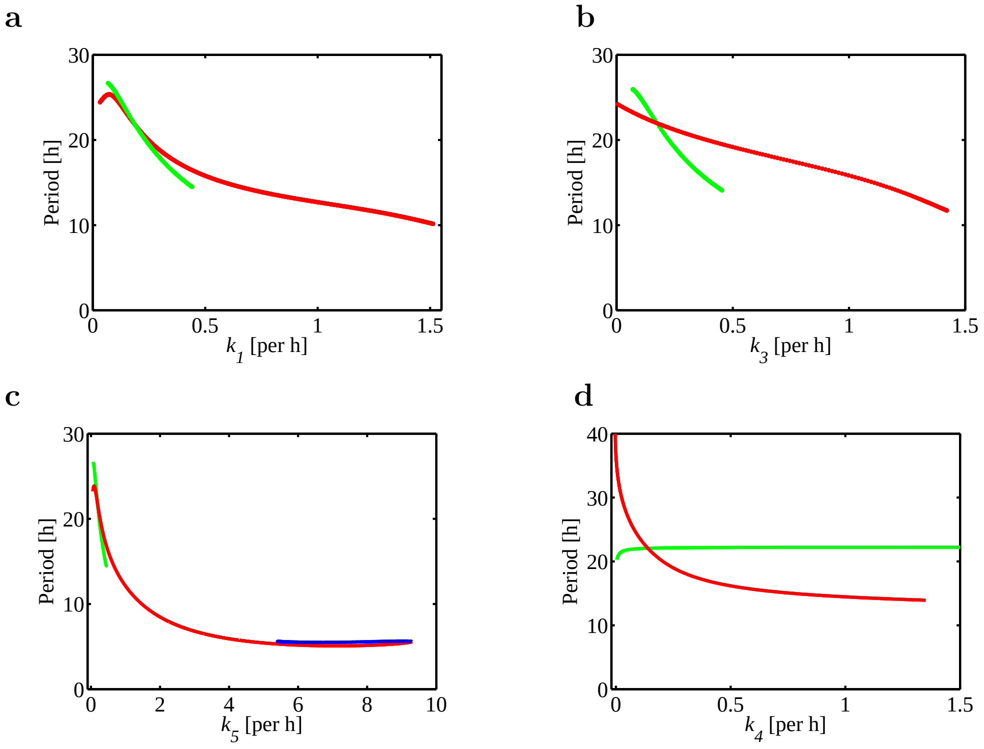

Figure S13. Period diagrams as functions of four different parameters. Green and red curves correspond to stable periodic orbits for Model 4 and Model 4', respectively. Blue curve corresponds to unstable periodic orbits for Model 4'.

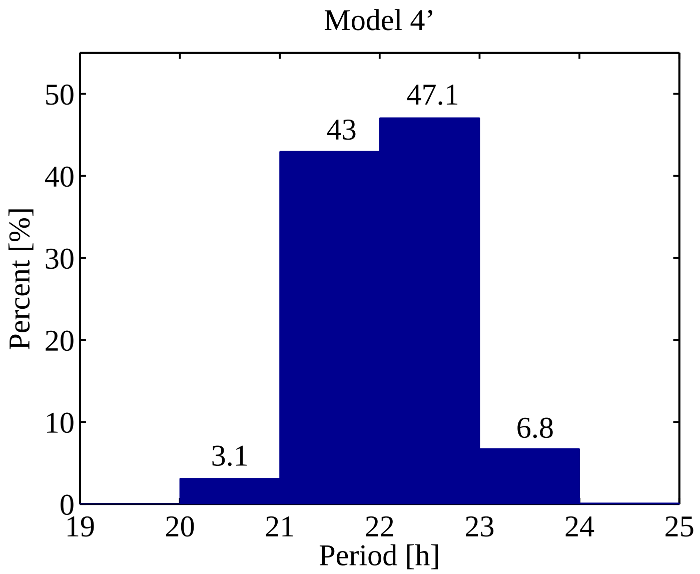

Figure S14. Histogram of period distribution obtained from 4000 random perturbations of parameter values in Model 4'.

Text S6. Stochastic models

Table S5 illustrates the stochastic version of Model 3. Four reactions and their corresponding propensities in the system are listed in the second and third columns, respectively. The last column displays the change in the number of molecules as a result of each reaction.

Table S5. Stochastic version of Model 3.

Reaction number	Reaction	Propensity of reaction	Transition
1	$\Phi \to M$	$w_1 = \dfrac{N\nu}{1+(P/(NK_a))^m}$	$M \to M - 1$
2	$M \to \Phi$	$w_2 = k_1 M$	$M \to M + 1$
3	$M \to M + P$	$w_3 = k_2 M$	$P \to P + 1$
4	$P \to \Phi$	$w_4 = \dfrac{k_3 P}{1+(P/(NK_b))^n}$	$P \to P - 1$

Figure S15 illustrates phase portraits of both deterministic and stochastic models in phase plane. The solid curves in red represent the limit cycle of deterministic models and the fluctuating curves around the limit cycle represent stochastic trajectories in Model 1 (top panel) and Model 4 (bottom panel). As expected, for the lower value of $N = 10$, the stochastic trajectories deviate considerably from the limit cycle of the ODEs due to a large fluctuation. As the volume size increases, the fluctuation of the stochastic oscillations decreases, that is, the system becomes more insensitive to molecular noise.

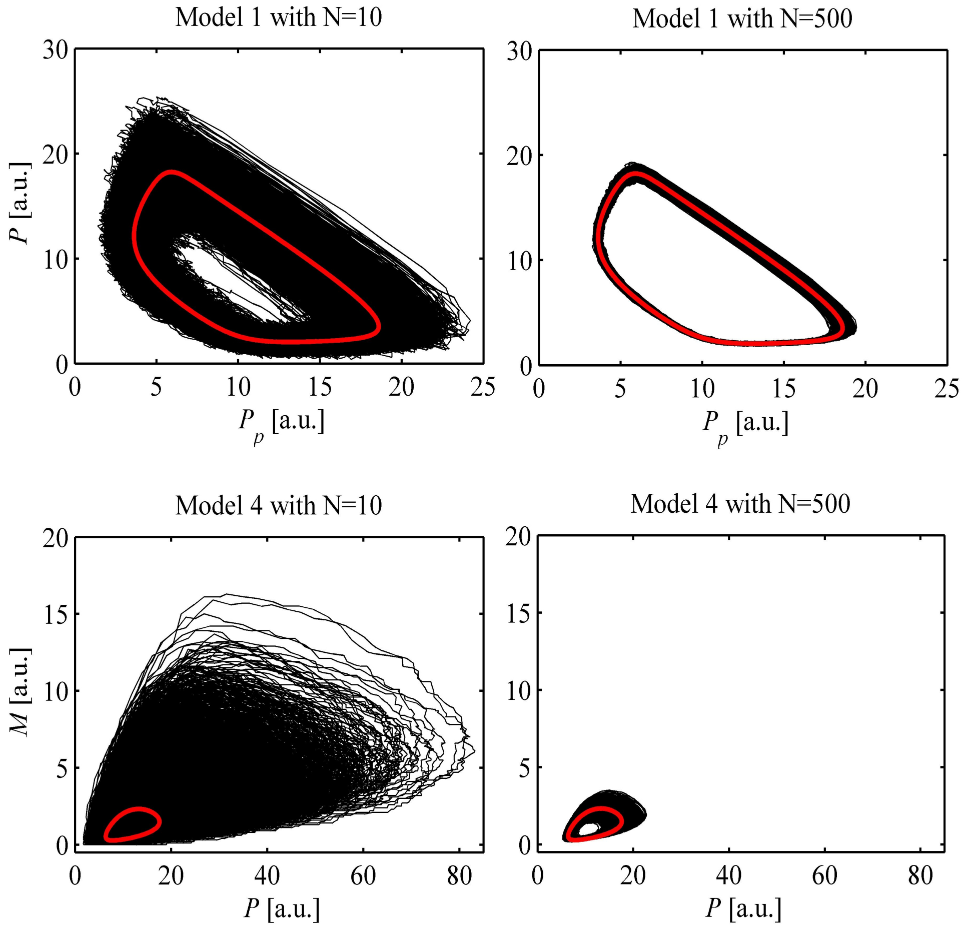

Figure S15. Limit cycles of the deterministic models and their corresponding stochastic oscillations for Models 1 and 4 when $N = 10$ and $N = 500$. Each panel shows the limit cycle (red) and stochastic trajectories (black) of protein (P), phosphorylated protein (P_p), and mRNA (M) in phase plane.

Figure S16 shows time evolution of autocorrelation functions of $Z_P^N(t)$ for different values of the volume factor in Model 5. This figure also illustrates the effect of molecular noise as N varies. Autocorrelations are computed with 4000 realizations of the stochastic model. The starting time of autocorrelations is taken at $t = 250$ h after the transient behavior of chemical components disappears. As shown in figure, the envelope of autocorrelations decreases slowly with the increasing volume factor. For the value of $N = 500$, for example, oscillations of the system are damped slowly. However, when N gets smaller, correlations disappear more rapidly because of the higher impact of fluctuations on the periodicity of the system. This indicates that how fast autocorrelation function is damped can be a measure of the robustness of the oscillatory systems [3,4]. Similar behaviors are observed in other models as well (data not shown).

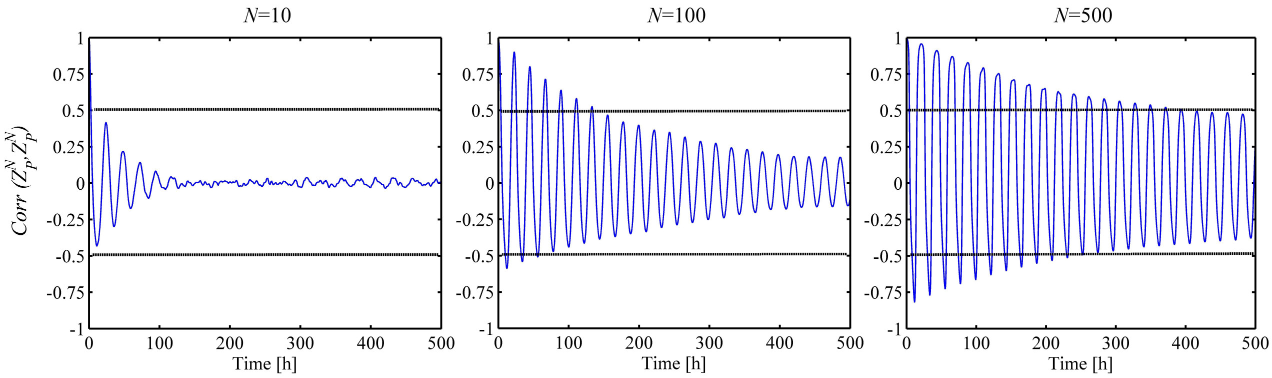

Figure S16. Time evolution of autocorrelation functions for different values of the volume factor N in Model 5. Each panel shows an autocorrelation function of $Z_P^N(t)$, where P represents protein. The autocorrelation is calculated over time duration $[250h, 750h]$, corresponding to ∼23 cycles.

Figure S17 illustrates the magnitude spectrum of protein P of Model 2, when the volume factor is set equal to either $N = 10$ (left panel) or $N = 100$ (right panel). Each panel illustrates the magnitude spectrum in frequency from the ODE model (dashed line) and the mean value of the magnitude spectrum in frequency obtained from 4000 individual realizations of stochastic simulations (solid line), whose standard deviation is represented by a shaded region around the mean value. The major frequency of Model 2 is around 0.045 Hz, which is equivalent approximately to 22 hours of period. Minor frequencies appear as multiples of $\sim$ 22 hours because of limit cycle oscillations of the ODE model. As seen in Figure 3, the discrepancy between the magnitude spectra of the deterministic and stochastic models is clearly reduced as N increases. Our simulation results confirm that the volume factor controls the number of molecules and thus the amplitude of fluctuations around the deterministic limit cycle depends on the number of molecules in the system.

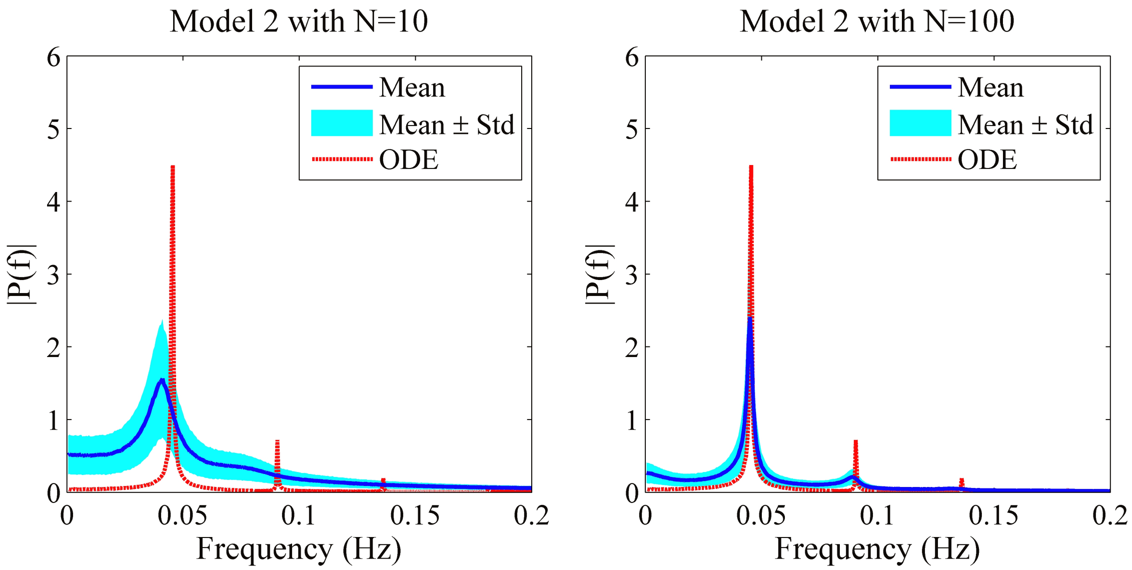

Figure S17. Magnitude spectrum of protein P of Model 2 with two different volume factors. The left and right panels show magnitude spectra of protein P when $N = 10$ and $N = 100$, respectively. Each panel illustrates the magnitude spectrum in frequency from the ODE model (dashed line) and the mean value of magnitude spectrum in frequency obtained from 4000 individual realizations of stochastic simulations (solid curve), whose standard deviation is represented by a shaded region around the mean value.

References

1. Guttman, R., Lewis, S. & Rinzel, J. Control of repetitive firing in squid axon membrane as a model for a neuroneoscillator. *The journal of physiology* **305**: 377-395 (1980).

2. Holden, A.V. Hopf bifurcation and the repetitive activity of excitable cells. In Mathematics in Biology and Medicine (eds V. Capasso, E. Grosso & S.L. Paveri-Fontana) 335-340 (Springer- Verlag, 1985).

3. Gonze, D., Halloy, J., & Gaspard, P. Biochemical clocks and molecular noise: Theoretical study of robustness factors. *The journal of chemical physics* **116**:10997-11010 (2002).

4. Gonze, D. & Goldebeter, A. Circadian rhythms and molecular noise. *Chaos* **16**(2): 026110 (2006).
